# NUP62 alleviates senescence and promotes the stemness of human dental pulp stem cells via NSD2-dependent epigenetic reprogramming

**DOI:** 10.1038/s41368-025-00362-y

**Published:** 2025-04-17

**Authors:** Xiping Wang, Li Wang, Linxi Zhou, Lu Chen, Jiayi Shi, Jing Ge, Sha Tian, Zihan Yang, Yuqiong Zhou, Qihao Yu, Jiacheng Jin, Chen Ding, Yihuai Pan, Duohong Zou

**Affiliations:** 1 School and Hospital of Stomatology, Wenzhou Medical University, Wenzhou, China; 2 Department of Orthodontics, Shanghai Ninth People’s Hospital, Shanghai Jiao Tong University School of Medicine; College of Stomatology, Shanghai Jiao Tong University; National Center for Stomatology; National Clinical Research Center for Oral Diseases; Shanghai Key Laboratory of Stomatology, Shanghai, China; 3 Department of Oral Surgery, Shanghai Ninth People’s Hospital, College of Stomatology, Shanghai Jiao Tong University School of Medicine; National Clinical Research Center for Oral Diseases; Shanghai Key Laboratory of Stomatology and Shanghai Research Institute of Stomatology, Shanghai, China; 4 State Key Laboratory of Genetic Engineering, School of Life Sciences, Human Phenome Institute, Fudan University, Shanghai, China; 5 Touro College of Dental Medicine, New York Medical College, New York, USA; 6 Department of Endodontics, School and Hospital of Stomatology, Wenzhou Medical University, Wenzhou, China

**Keywords:** Ageing, Regeneration, Reprogramming

## Abstract

Stem cells play a crucial role in maintaining tissue regenerative capacity and homeostasis. However, mechanisms associated with stem cell senescence require further investigation. In this study, we conducted a proteomic analysis of human dental pulp stem cells (HDPSCs) obtained from individuals of various ages. Our findings showed that the expression of NUP62 was decreased in aged HDPSCs. We discovered that NUP62 alleviated senescence-associated phenotypes and enhanced differentiation potential both in vitro and in vivo. Conversely, the knocking down of NUP62 expression aggravated the senescence-associated phenotypes and impaired the proliferation and migration capacity of HDPSCs. Through RNA-sequence and decoding the epigenomic landscapes remodeled induced by NUP62 overexpression, we found that NUP62 helps alleviate senescence in HDPSCs by enhancing the nuclear transport of the transcription factor E2F1. This, in turn, stimulates the transcription of the epigenetic enzyme NSD2. Finally, the overexpression of NUP62 influences the H3K36me2 and H3K36me3 modifications of anti-aging genes (HMGA1, HMGA2, and SIRT6). Our results demonstrated that NUP62 regulates the fate of HDPSCs via NSD2-dependent epigenetic reprogramming.

## Introduction

The life expectancy of humans has steadily increased over the past century, resulting in an increased prevalence of age-related diseases, such as neurodegenerative diseases, diabetes mellitus, osteoporosis, and osteoarthritis.^1^ Age-related diseases not only influence the quality of life for older individuals but also impose a significant financial burden on society. Recent studies on aging have focused on elucidating common features, such as telomere dysfunction, genomic instability, epigenetic alterations, impaired autophagy, cellular senescence, and stem cell exhaustion that influence the process of aging.^2,3^ Understanding the aging process will help to identify appropriate therapeutic strategies for delaying or preventing age-related disease progression.

Stem cells play a critical role in maintaining of tissue regenerative capacity and homeostasis.^4,5^ Adult stem cell aging and exhaustion play a significant role in the overall aging of adult organisms.^3^ Human dental pulp stem cells (HDPSCs), which originate from the ectomesenchyme of the cranial neural crest,^6–8^ are a specific type of adult stem cells. HDPSCs preserve their regenerative capacity in adult teeth and contribute to the maintenance of homeostasis in dental stromal tissue.^9^ HDPSCs, which are isolated from permanent teeth, are readily accessible Mesenchymal Stem Cells (MSCs) that can meet the needs of recipients of various ages. Additionally, HDPSCs demonstrate an age-dependent decrease in proliferation and multilineage differentiation.^10–12^ This characteristic makes them a reliable model for studying mechanisms associated with senescence. It has been previously demonstrated that the downregulation of ROR2 accelerated the senescence of DPSCs through the activation of the MSX2/NSUN2/p21 axis.^13^ Yang R.L. et al. reported that serine metabolism regulates the senescence of DPSCs by influencing DNA methylation of p16.^10^ Furthermore, mettl3-mediated m^6^A modification influenced the cell cycle progression of DPSCs.^14^ The molecular mechanism underlying DPSC senescence requires further investigation.

Nuclear pore complexes (NPCs), comprised of approximately 30 nucleoporin proteins (Nups),^15^ not only mediate nucleocytoplasmic transport but also play a crucial role in genome organization and cellular homeostasis.^16–18^ Interestingly, the dysfunction of NPCs and abnormalities in specific nucleoporins have been associated with age-related diseases.^19–21^ This suggests that the composition and function of NPCs may play a significant role in the aging process. NPCs regulate gene expression by actively participating in the selective import of transcription factors, as well as chromatin remodeling and histone modification.^22^ NUP62, located in the central avenue of NPC, plays a vital role in regulating selective transport between the nucleus and cytoplasm.^21^ Researchers have conducted extensive screenings to identify Nups associated with the pathogenesis of Huntington’s disease. They discovered that NUP62, NUP88, and RanGAP1 are significant pathogenic factors of Huntington’s disease.^23^ However, the contribution of Nup-based mechanisms to age-related diseases remains to be investigated. Additionally, there is limited information regarding the changes in Nups during the aging of HDPSCs.

Here, we examined the proteomic characteristics of HDPSC samples collected from individuals of various ages. We found that the expression of NUP62 was decreased in aged HDPSCs. Upregulation of NUP62 expression in HDPSCs alleviated senescence-associated phenotypes and enhanced differentiation potential both in vitro and in vivo. Additionally, overexpression of NUP62 facilitated the nuclear transport of E2F1, which then binds to the promoter region of NSD2 to enhance its expression. Notably, NUP62 overexpression influenced the H3K36me2 and H3K36me3 modifications of anti-aging genes (HMGA1, HMGA2, and SIRT6). Conversely, when NUP62 was knocked down, NSD2 expression decreased, resulting in a global reduction of H3K36me2 and H3K36me3 levels.

## Results

### The expression of NUP62 was decreased with age

**Fig. 1:**
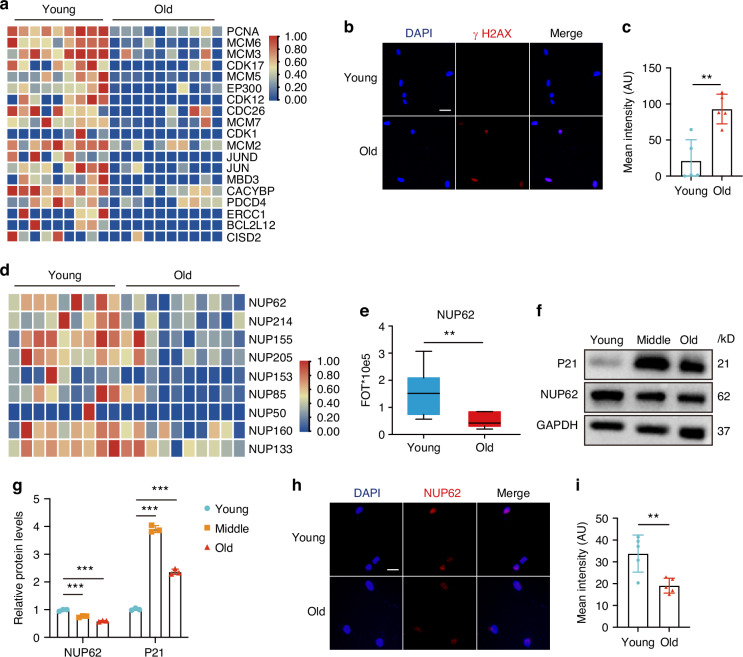
NUP62 mRNA and protein levels are decreased in aged human dental pulp stem cells (HDPSCs). **a** Heatmap of signature proteins that were differentially expressed in HDPSCs isolated from different donors (>1.5-fold, *t* test, *p* < 0.05). **b**, **c** Immunofluorescence staining of γH2AX and quantification of fluorescence intensity (*n* = 5). Scale bar, 50 μm. **d** Heatmap of Nups expression in HDPSCs isolated from different donors (> 1.5-fold, t test, *p* < 0.05). **e** Boxplot of NUP62 levels in HDPSCs isolated from different donors. **f, g** Western blot analyses of NUP62 and P21 expression with age. **h**, **i** Immunofluorescence staining of NUP62 and quantification of fluorescence intensity (*n* = 5). Scale bar, 50 μm. **P < 0.01 and ***P < 0.001

HDPSCs were isolated from donors aged 16 to 70 years. The isolated and cultured HDPSCs exhibited high expression of Mesenchymal Stem Cell (MSC) markers, including CD73, CD90, CD29, CD44, and CD146. Additionally, these cells demonstrated the ability to differentiate into osteogenic, adipogenic and chondrogenic lineages (Fig. S1a–d). A proteomic analysis was conducted on HDPSC samples isolated from subjects of various ages to identify proteins that vary with age. A heatmap from the proteomic analysis showed that the expression of cell cycle-related genes, such as PCNA, MCM6, MCM3, CDK17, CDK12, MCM5, MBD3, BCL2L12, and CACYBP decreased with increasing age (Fig. 1a). Immunofluorescence staining for γH2AX in young (Y)-HDPSCs and old (O)-HDPSCs revealed that γH2AX expression increased with age (Figs. 1b and c). A heatmap of the proteomic analysis indicated a decline in the expression of Nups, particularly a significant downregulation of NUP62 expression (Fig. 1d). The results showed that the NUP62 expression was decreased with age (Fig. 1e). Western blot analysis and RT‒PCR also supported these findings (Fig. 1f, g and fig. S2a). The top 20 genes identified in the proteomic analysis were presented in Fig. S3a. Additionally, the expression of senescence-associated genes (P16 and P21) and senescence-associated secretory phenotype (SASP) genes (interleukin-6 and interleukin-8) increased with age (Fig. S3b). Our studies showed that both the proliferation and osteogenic differentiation capacity of HDPSCs decreased with age, while the percentage of cells positively stained for senescence-associated beta-galactosidase (SA β-gal) increased with age (Fig. S3c–f). Immunofluorescence staining further validated the age-related decrease in NUP62 expression (Fig. 1h, i). Following the induction of replicative senescence in HDPSCs through cell passaging, Western blot and RT‒PCR analyses demonstrated that the expression level of NUP62 decreased with an increasing number of HDPSC passages (Fig. S2b–d). As expected, the mRNA levels of P16 and P21 were found to be elevated (Fig. S2d). These results suggested that NUP62 expression was correlated with HDPSC senescence, indicating that NUP62 may play a critical role in regulating the senescence of HDPSCs.

### NUP62 upregulation alleviated senescence and promoted the differentiation potential of O-HDPSCs

**Fig. 2:**
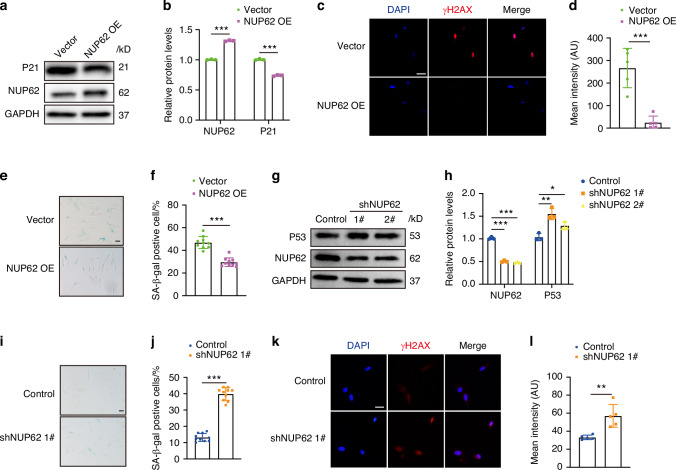
NUP62 regulates human dental pulp stem cell (HDPSC) senescence. **a**, **b** Western blotting for NUP62, P21, and GAPDH (loading control) was performed using lysates from HDPSCs transfected with Lentiviral vector and overexpressing NUP62. **c**, **d** Immunofluorescence staining of γH2AX and quantification of fluorescence intensity (*n* = 5). Scale bar, 50 μm. **e,** **f** Representative images of senescence-associated β-galactosidase (SA β-gal) staining of O-HDPSCs and quantification of positive cells (*n* = 10). Scale bar, 50 μm. **g, h** Western blot analyses of NUP62, P53, and GAPDH expression in HDPSCs transfected with empty lentiviral control or in which NUP62 expression was via lentiviral delivery of shRNAs targeting 2 different sequences (#1, #2). **i,** **j** Representative images of SA β-gal staining and quantification of positive cells (*n* = 10). Scale bar, 50 μm. **k,** **l** Immunofluorescence staining of γH2AX and quantification of fluorescence intensity (*n* = 5). Scale bar, 50 μm. *P < 0.05, **P < 0.01 and ***P < 0.001

To verify the role of NUP62 in both cellular senescence and the impaired differentiation potential associated with aging, we overexpressed NUP62 in O-HDPSCs using lentiviral infection. We then assessed the senescence-associated phenotypes and differentiation potential of these cells. The efficiency of NUP62 overexpression was confirmed through Western blotting and RT‒PCR (Fig. 2a, b and S4a). We observed a decrease in γH2AX, a marker of nuclear DNA double-strand breaks associated with senescence, in O-HDPSCs that overexpressing NUP62 (Fig. 2c, d). The mRNA levels of key senescence markers such as P16 and P53, as well as the senescence-associated metalloprotease MMP13, interleukin-8, and interleukin-1α were also lower in O-HDPSCs overexpressing NUP62 (Fig. S4a). Additionally, the percentage of SA β-gal-positive cells was significantly reduced in the O-HDPSCs with NUP62 overexpressing compared to control HDPSCs (Fig. 2e, f). Furthermore, overexpression of NUP62 improved the migration ability of O-HDPSCs (Fig. S4b, c).

We have induced the senescence of HDPSCs through cell passaging (P). Subsequently, we overexpressed NUP62 in the (12P) aged HDPSCs to investigate its role in regulating HDPSC senescence. The overexpression of NUP62 in the (12P) aged HDPSCs resulted in a decrease in the expression of P21 and a reduction in the percentage of SA β-gal-positive cells (Fig. S 5a-d). Our findings showed that overexpression of NUP62 effectively alleviated the senescence of O-HDPSCs.

We conducted further investigations to determine whether NUP62 overexpression enhances the differentiation potential of HDPSCs. After treatment with an osteogenic differentiation medium for 14 days, the expression of osteogenic differentiation-related genes, including RUNX2 and OSX, as well as odontogenic differentiation-related genes, such as DMP1 and DSPP, were significantly elevated in O-HDPSCs overexpressing NUP62 (Fig. S4d, e). Compared to control HDPSCs, HDPSCs that overexpress NUP62 demonstrated a significantly improved capacity of osteogenic differentiation, as evidenced by Alizarin Red staining (Fig. S4f, g). Moreover, the mRNA levels of key osteogenic differentiation-related genes (ALP and OSX) and odontogenic differentiation-related genes (DMP1 and DSPP) were noticeably increased in HDPSCs overexpressing NUP62 (Fig. S4h).

After 9 days of induction with a neurogenic medium, Western blot analysis revealed that the expression levels of GFAP, SOX2, and MAP2 genes were significantly elevated in O-HDPSCs overexpressing NUP62 (Fig. S4i, j). The RT‒PCR results showed that the mRNA levels of MAP2, SOX2, S100B, and βIII tubulin were higher in NUP62-overexpressing O-HDPSCs compared to the control O-HDPSCs (Fig. S4k). These results demonstrated that the overexpression of NUP62 enhanced the multilineage differentiation potential of O-HDPSCs, including osteogenic, odontogenic, and neurogenic differentiation.

### NUP62 knockdown promoted Y-HDPSC senescence and impaired the differentiation potential of Y-HDPSCs

We knocked down the expression of NUP62 in Y-HDPSCs and assessed the senescence-associated phenotypes. The efficiency of the knockdown was confirmed using Western blotting and RT‒PCR (Fig. 2g, h and fig. S6a). We observed a significant increase in the expression of P21 in NUP62-depleted Y-HDPSCs (Fig. S6b). Additionally, the mRNA levels of SASP genes, such as MMP13, interleukin-8, and interleukin-1α, were also elevated in NUP62-depleted Y-HDPSCs (Fig. S6b). Furthermore, the proliferation and migration capacities of NUP62-depleted Y-HDPSCs were significantly decreased (Fig. S6c–e). The percentage of SA β-gal-positive cells in NUP62-depleted Y-HDPSCs was significantly higher compared to the control group (Fig. 2i, j). Immunofluorescence staining revealed that γH2AX was significantly elevated in Y-HDPSCs with NUP62 depletion (Fig. 2k, l). These results indicated that the knockdown of NUP62 promoted HDPSC senescence.

We next investigated whether NUP62 knockdown impaired the differentiation potential of HDPSCs. After treating the cells with an osteogenic differentiation medium for 7 days, we observed that the mRNA levels of key osteogenic differentiation-related genes (ALP and OSX) and odontogenic differentiation-related genes (DMP1 and DSPP) were significantly decreased in the NUP62 knockdown HDPSCs compared to those infected with lentiviral vector (Fig. S6f). Additionally, the osteogenic differentiation capacity of HDPSCs with NUP62 knockdown was impaired, as indicated by Alizarin Red staining (Figs. S6g, h). After 9 days of induction with a neurogenic medium, RT‒PCR results showed that the mRNA levels of SOX2, S100B, GAP43, and NESTIN were lower in Y-HDPSCs with NUP62 knockdown compared to the control O-HDPSCs (Fig. S6i).

### Overexpression of NUP62 promotes the osteogenic differentiation of O-HDPSCs in vivo

**Fig. 3:**
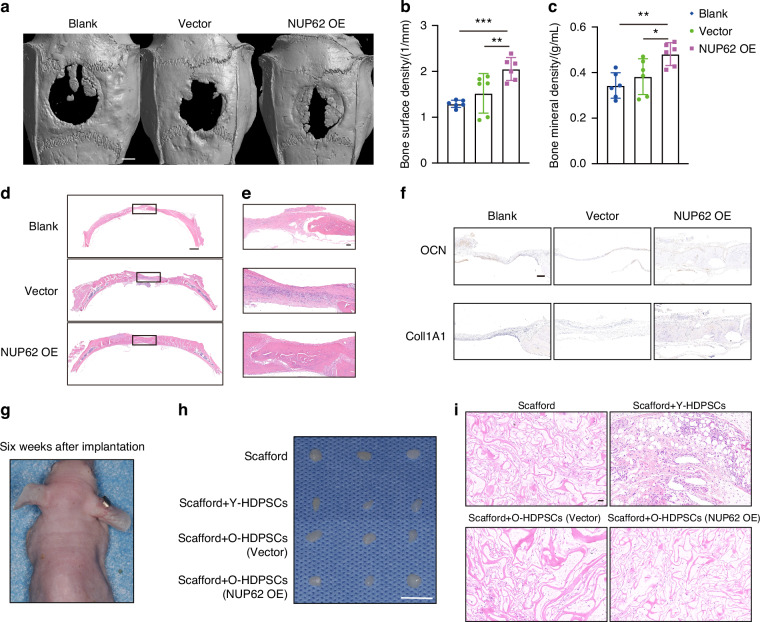
Overexpression of NUP62 promotes the osteogenic differentiation of O-human dental pulp stem cell (HDPSCs) in vivo. **a** Representative images of micro-CT reconstructions of rat calvarial bone defects at 8 weeks. Scale bar, 2 mm. **b** Quantitative micro-CT analysis of bone surface density (BSD, 1/mm) (*n* = 6). **c** Quantitative micro-CT analysis of bone mineral density (BMD, g/cc) (*n* = 6). **d** Representative images of H&E-stained rat calvarial bone defects. Scale bar, 1 mm. **e** Representative images of H&E-stained rat calvarial bone defects. Scale bar, 100 μm. **f** Representative images of immunohistochemically stained rat calvarial bone defects. Scale bar, 200 μm. **g** Representative image of nude mice six weeks after xenografting. **h** Sponges loaded with Y-HDPSCs or O-HDPSCs transfected with an empty lentiviral vector or overexpressing NUP62 were subcutaneously transplanted for xenografting (*n* = 3). Scale bar, 1 cm. **i** Representative images of H&E-stained xenografts. Scale bar, 50 μm. *P < 0.05, **P < 0.01 and ***P < 0.001

As shown by three-dimensional micro-CT imaging, Group 3 (the scaffold + O-HDPSCs transfected with NUP62 overexpressed) exhibited markedly greater bone regeneration (Fig. 3a). Additionally, quantitative micro-CT analysis showed that the bone surface density in Group 3 was twice that in Group 1 (the scaffold without HDPSCs) (Fig. 3b). Quantitative analysis revealed that the bone mineral density (BMD) in Group 3 was greater than that in the other two groups (*P* < 0.05) (Fig. 3c). In Group 1, the defect area was primarily covered by fibrous tissue, with minimal new bone formation observed (Fig. 3d, e). Similarly, Group 2 (the scaffold + O-HDPSCs transfected with an empty vector) exhibited little new bone formation, and most of its defect area was covered by fibrous tissue (Fig. 3d, e). In contrast, Group 3 displayed significant new bone formation, covering nearly all the defect areas (Fig. 3d, e). Immunohistochemistry results revealed increased expression levels of osteogenic biomarkers, including COL1A1 and OCN, in Group 3 (Fig. 3f), suggesting that NUP62 overexpression in O-HDPSCs enhances bone regeneration. Xenografts were established with Y-HDPSCs and O-HDPSCs transfected with empty vector or overexpressing NUP62. The O-HDPSCs overexpressing NUP62 did not increase in tumor volume compared to those transfected with empty vector, (Fig. 3g, h). Moreover, H&E staining of xenografts showed no sign of abnormal cell proliferation (Fig. 3i).

### NUP62 overexpression ameliorates senescence-associated hallmarks of O-HDPSCs by upregulating NSD2

**Fig. 4:**
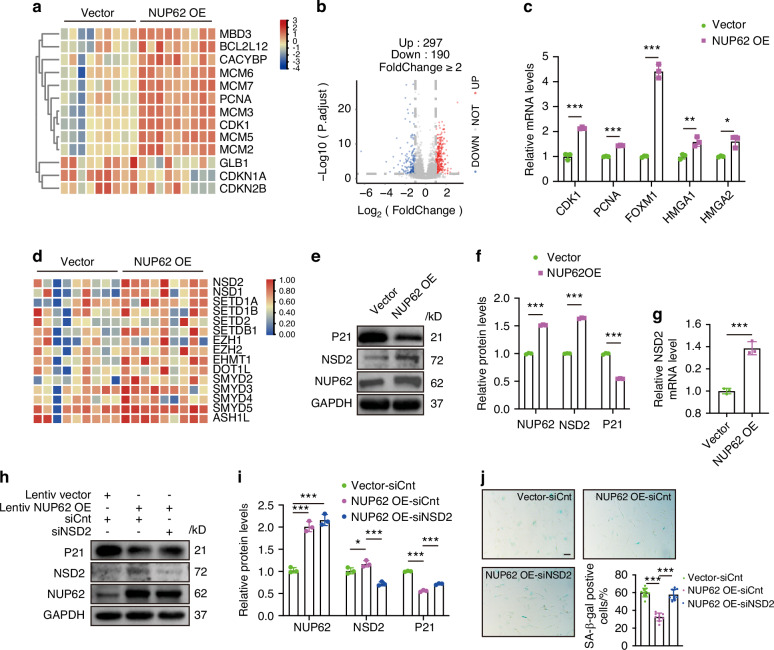
Overexpression of NUP62 ameliorates senescence-associated hallmarks of O-human dental pulp stem cells (HDPSCs) via NSD2 upregulation. **a** Heatmap of differentially expressed genes in HDPSCs with and without NUP62 overexpression (*n* = 9). **b** Volcano plot indicating the differential expression of genes in HDPSCs with and without NUP62 overexpression. **c** Quantitative RT‒PCR results of representative genes (*n* = 3). **d** Heatmap of representative epigenetics-related genes in HDPSCs with and without NUP62 overexpression (*n* = 9). **e**, **f** Western blot analyses of NUP62, P21, NSD2, and GAPDH expression in HDPSCs transfected with lentiviral vector or overexpressing NUP62. **g** Quantitative RT‒PCR results for NSD2 (*n* = 3). **h,** **i** Western blot analyses of NUP62, P21, NSD2 and GAPDH expression in HDPSCs transfected with a lentiviral vector or NUP62 overexpression lentivirus and control siRNA (NC) or NSD2 siRNA. **j** Representative images of SA β-gal staining and quantification of positive cells (*n* = 10). Scale bar, 50 μm. *P < 0.05, **P < 0.01 and ***P < 0.001

RNA sequencing (RNA-seq) was performed to analyze the transcriptome profiles of HDPSCs transfected with an empty vector and those overexpressing NUP62. The results indicated that the expression of cell cycle-related genes (MCM6, MCM7, PCNA, MCM3, CDK1, MCM5, MCM2, MBD3, BCL2L12 and CACYBP) was elevated in HDPSCs overexpressing NUP62. Conversely, the expression of senescence-related genes (GLB1, CDKN1A, and CDKN2B) was decreased (Fig. 4a). SA β-galactosidase, a cellular senescence marker, is encoded by GLB1.^24^ GLB1 expression levels can serve as an indicator of organ dysfunction in vivo.^25^ A total of 487 differentially expressed genes (DEGs) (log2-fold change > 2 and *P* < 0.05) were identified. Among these genes, 297 were found to be upregulated while 190 were downregulated (Fig. 4b). Our RT‒PCR analysis confirmed the RNA-seq findings (Fig. 4c). Overexpression of NUP62 upregulated the mRNA levels of CDK1, PCNA, FOXM1, HMGA1 and HMGA2 (Fig. 4c). Given the role of histone modifications in aging, we further investigated changes in the expression of histone-modifying kinases. Notably, NSD2 expression was significantly increased in HDPSCs overexpressing NUP62 (Fig. 4d). This upregulation of NSD2 was confirmed through both Western blot and RT‒PCR analyses (Fig. 4e–g). In contrast, the mRNA levels of NSD2, CDK1, PCNA, and HMGA1 decreased in aged HDPSCs (Fig. S3b). To further investigate whether NUP62 alleviates HDPSC senescence by regulating NSD2, we found that NUP62 upregulation led to a decrease in P21 expression and a reduction in the percentage of SA β-gal-positive cells. Notably, these effects were reversed following siRNA‒mediated NSD2 knockdown (Fig. 4h–j). The efficiency of NSD2 knockdown was evaluated using Western blot and RT‒PCR (Fig. S7a–c). Furthermore, the expression of NSD2 was significantly lower in NUP62-depleted HDPSCs compared to control HDPSCs (Fig. S8a, b).

### NUP62 mediates epigenetic reprogramming by upregulating the global levels of H3K36me2 and H3K36me3

**Fig. 5:**
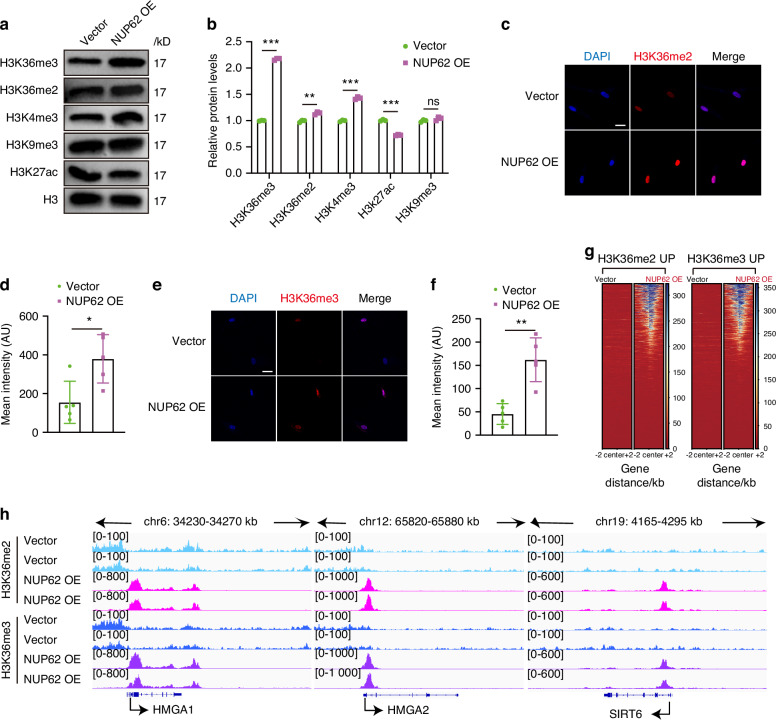
NUP62 mediates epigenetic reprogramming by upregulating the global levels of H3K36me2 and H3K36me3. **a**, **b** Western blot analyses of H3K36me2, H3K36me3, H3K27ac, H3K4me3, and H3K9me3 in human dental pulp stem cells (HDPSCs) transfected with lentiviral vectors or overexpressing NUP62. **c**, **d** Immunofluorescence staining and quantification of H3K36me2, respectively (*n* = 5). Scale bar, 50 μm. **e,** **f** Immunofluorescence staining and quantification of H3K36me3, respectively (*n* = 5). Scale bar, 50 μm. **g** Heatmap of global H3K36me2 and H3K36me3 CUT&Tag-seq signals from HDPSCs transfected with empty lentiviral vector or NUP62 overexpression lentivirus (*n* = 2). **h** Gene track view of normalized bigWig reads at the promoters of SIRT6, HMGA1 and HMGA2 in HDPSCs transfected with a lentiviral vector or overexpressing (*n* = 2). *P < 0.05, **P < 0.01 and ***P < 0.001

We next sought to investigate whether NUP62 regulates histone modification. Western blot analysis revealed that NUP62 overexpression led to an increase in the global levels of H3K36me2 and H3K36me3 (Fig. 5a, b), which was confirmed by immunofluorescence staining (Fig. 5c–f). Conversely, knockdown of NUP62 resulted in a decrease in the global levels of H3K36me2 and H3K36me3 (Fig. S8c, d).

To determine the genome-wide binding patterns of H3K36me2 and H3K36me3, we performed cleavage under target and tag (CUT&Tag) sequencing in HDPSCs transfected with either empty vector or overexpressing NUP62. The analysis revealed distinct global and genome-wide patterns of H3K36me2 and H3K36me3 modifications in HDPSCs overexpressing NUP62 compared to those with the empty vector (Fig. 5g). We found greater enrichment of H3K36me2 and H3K36me3 in HDPSCs overexpressing NUP62 (Fig. 5g). A total of 1818 genes were found to be upregulated in HDPSCs with overexpressing NUP62, of which 536 genes (29.5%) overlapped with elevated H3K36me2 binding peaks in these cells. Furthermore, 499 of these genes (27.4%) overlapped with elevated H3K36me3 binding peaks in HDPSCs overexpressing NUP62 (Fig. S9a). Among the identified genomic regions, several anti-aging genes, including high mobility group AT-hook 1 (HMGA1), high mobility group AT-hook 2 (HMGA2) and SIRT6, exhibited elevated H3K36me2 and H3K36me3 binding peaks in HDPSCs overexpressing NUP62 (Fig. 5h). ChIP-qPCR analyses confirmed that NUP62 overexpression influenced the H3K36me2 and H3K36me3 epigenetic modification of anti-aging genes (HMGA1, HMGA2 and SIRT6) (Fig. S9b).

### NUP62 affects NSD2 transcription by facilitating the nuclear transport of transcription factor E2F1

**Fig. 6:**
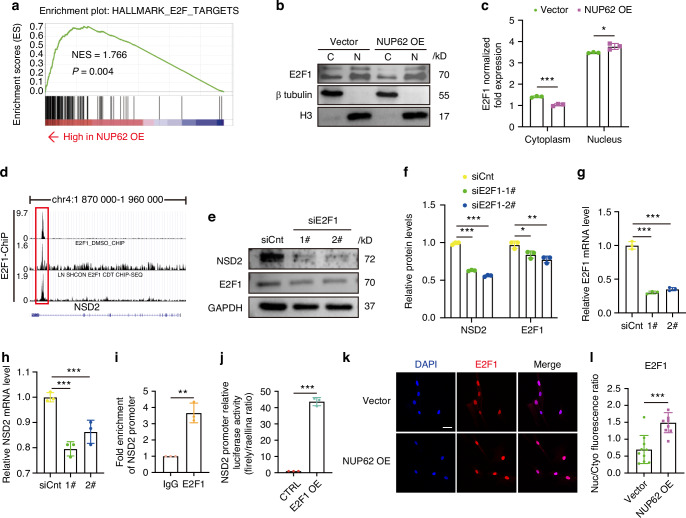
NUP62 affects NSD2 transcription by facilitating the nuclear transport of the transcription factor E2F1. **a** Gene set enrichment analysis (GSEA) identified distinct induction of the target gene signatures of the transcription factor E2F1 in human dental pulp stem cells (HDPSCs) with and without NUP62 overexpression. **b** Western blot analysis of E2F1 levels in the nucleoplasm and cytoplasm of HDPSCs transfected with the lentiviral vector or overexpressing NUP62. **c** Quantitative analysis of the E2F1 Western blotting results (*n* = 3). **d** Mapping of E2F1 ChIP-seq reads to the NSD2 genomic locus. The figure was obtained from the Cristrome database. **e, f** Western blot analyses of E2F1 and NSD2 in HDPSCs transfected with control or E2F1 siRNA. **g**, **h** mRNA levels of E2F1 and NSD2 in HDPSCs transfected with control or E2F1 siRNA (*n* = 3). **i** ChIP‒qPCR revealed that E2F1 binds to the promoter region of NSD2 (*n* = 3). **j** Luminescence analysis of 293 T cells following co-transfection with an empty vector or overexpressing E2F1, a promoter luciferase reporter (wild type), and a Renilla control (*n* = 3). **k** Representative images of immunofluorescence staining of E2F1. Scale bar, 50 μm. **l** Nuclear/Cytoplasmic fluorescence ratio of E2F1 (*n* = 10). *P < 0.05, **P < 0.01 and ***P < 0.001

Given that NUP62 alleviates HDPSC senescence via NSD2 regulation, we further analyzed the molecular mechanisms by which NUP62 regulates NSD2 expression. Gene set enrichment analysis (GSEA) revealed distinct signatures of E2F1 transcription factor target genes in HDPSCs overexpressing NUP62 compared to the control group (Fig. 6a).

**Fig. 7:**
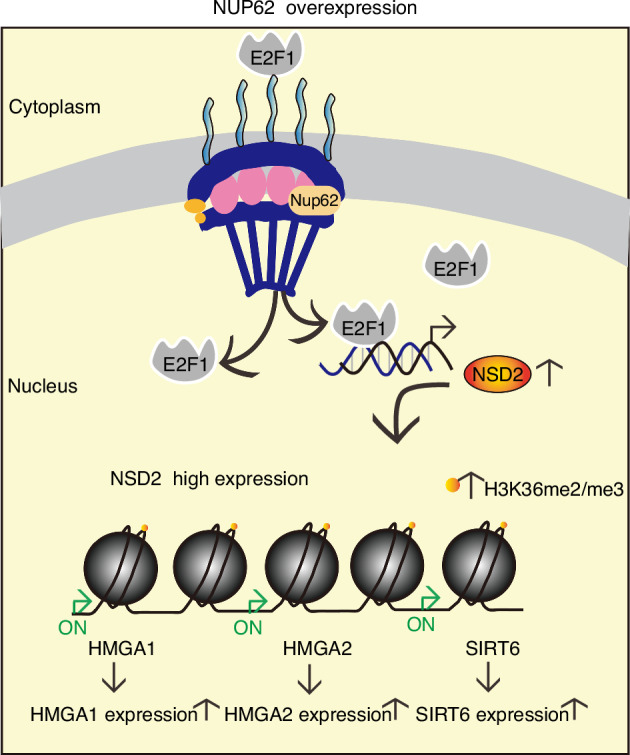
Schematic of mechanism by which NUP62 regulates senescence of HDPSCs

E2F1 is a transcription factor that is transported into the nucleus and participates in cellular proliferation, apoptosis, and differentiation.^26,27^ NUP62, a phenylalanine-and glycine-rich nucleoporin (FG-Nup), plays a vital role in nucleocytoplasmic transport.^21,28^ Next, we further analyzed the distribution of E2F1 in nuclear and cytoplasmic of HDPSCs that overexpress NUP62. Western blotting analysis of separate nuclear and cytoplasmic fractions from HDPSCs transfected with empty vector and those overexpressing NUP62 further revealed that the nuclear import of E2F1 was significantly higher in HDPSCs overexpressing NUP62 (Fig. 6b, c). The E2F1 binding site on the NSD2 gene promoter region showed significant overlap (Fig. 6d). To confirm that E2F1 regulate NSD2, we performed siRNA‒mediated knockdown of E2F1, which resulted in decreased NSD2 mRNA and protein levels (Fig. 6e–h). ChIP-qPCR further confirmed that E2F1 binds to the NSD2 promoter region (Fig. 6i). Luminescence analysis of 293 T cells co-transfection with an empty or a plasmid overexpressing E2F1, along with an NSD2 promoter luciferase reporter (wild type), and a Renilla luciferase reporter showed that E2F1 overexpression activated luciferase transcription from the NSD2 promoter reporter (Fig. 6j). The increase in the nuclear localization of E2F1 was further confirmed by immunofluorescence staining (Fig. 6k, l). Moreover, upregulation of NUP62 resulted in increased NSD2 expression, and this effect was reversed by E2F1 knockdown (Fig. S10a–c). Taken together, these results suggest that NUP62 modulates E2F1 nuclear localization, causing E2F1 to bind to the NSD2 promoter region and upregulate NSD2 transcription. Finally, overexpression of NUP62 was found to influence the H3K36me2 and H3K36me3 modifications of anti-aging genes (HMGA1, HMGA2, and SIRT6) (Fig. 7).

## Discussion

Stem cell aging is thought to be an important driver of organismal aging.^3^ The induction of stem cell rejuvenation is promising for ameliorating age-associated phenotypes in vivo.^29,30^ Elucidating the mechanisms through which stem cell aging occurs could help to develop new strategies for treating age-related diseases. Recent studies has shown that NPCs are associated with age-related diseases. However, it is still unclear whether Nups change in human stem cells during aging and whether these changes influence the aging of stem cells. Previous studies have linked NUP62 to neurodegenerative disorders such as ALS and FTLD, demonstrating that the overexpression of NUP62 can effectively rescue eye degeneration in flies.^30–32^ However, the changes in NUP62 expression in human stem cells and its role as a regulator of senescence have yet to be thoroughly investigated. Our study aims to examine how NUP62 expression changes with age and the mechanisms by which NUP62 regulates the renewal and differential capacity of HDPSCs.

Our results indicated that levels of NUP62 decreased with age. Overexpressing NUP62 ameliorated the senescence-associated phenotypes and enhanced the differentiation potential of O-HDPSCs. NUP62, a phenylalanine-glycine nucleoporin (FG-Nup), plays a critical role in regulating selective nucleocytoplasmic transport.^21,28,33^ Impaired nucleocytoplasmic transport is a key feature of aging and neurodegenerative diseases.^19,21,32,34^ Notably, our data revealed a significant interaction between the overexpression of NUP62 and E2F signaling activity. Specifically, we found that the upregulation of NUP62 enhances the nuclear transport of the transcription factor E2F1, which then binds to the promoter of the epigenetic enzyme NSD2 to enhance its transcription. However, it is still unclear whether the upregulation of NUP62 is associated with structural changes in NPCs. Additionally, it remains to be determined whether NUP62 facilitates the nuclear transport of the transcription factor E2F1 through a direct interaction or with assistance from the nuclear import receptor importin β.

We demonstrated that NUP62 influences age-related phenotypes by regulating NSD2. NSD2 is a methyltransferase responsible for di- and tri-methylation of histone H3 at lysine 36. As a chromatin regulator, NSD2 helps prevent the epigenomic remodeling changes associated with cellular senescence.^35,36^ The methylation of H3K36 by NSD2 is crucial for the development of adipose tissue and for spermatogenesis.^37,38^ A significant loss of epigenetic information is a key hallmark of aging.^39^ Moreover, H3K36 methylation plays a critical role in longevity in yeast and *Caenorhabditis elegans*.^40,41^ In chromatin, histone modifications play a critical role in regulating gene expression. Numerous studies have demonstrated that the enrichment of H3K36me2 and H3K36me3 in the body of genes is associated with transcriptional activation.^42,43^ Our data indicated that global levels of H3K36me2 and H3K36me3 increased following the overexpression of NUP62. Our results showed that the NUP62-mediated regulation of NSD2 expression primarily influenced the levels of H3K36me3, rather than H3K36me2 levels. This finding aligns with the more significant decrease in H3K36me3 levels compared to H3K36me2 levels observed in the pachytene spermatocytes and round spermatids of *Nsd2* conditional knockout mice.^38^ It is widely accepted that SETD2 is the enzyme responsible for all H3K36 trimethylation.^44^ However, our RNA-seq results revealed no significant change in SETD2 expression in HDPSCs overexpressing NUP62. Therefore, the relationship between NSD2 and H3K36me3 requires further investigation.

Our data revealed a greater enrichment of H3K36me2 and H3K36me3 modifications in HDPSCs that overexpress NUP62. This suggests that the overexpression of NUP62 may help restore lost epigenetic information. Additionally, an analysis of genes with increased binding peaks of H3K36me2/3 in HDPSCs overexpressing NUP62 identified several anti-aging genes, including HMGA1, HMGA2, and SIRT6. HMGA1 is highly expressed in embryonic stem cells, which are characterized by a strong capacity for proliferation.^45,46^ Furthermore, research has shown that the overexpression of the *HMGA1P6* pseudogene in mouse embryos promotes longevity in mice.^47^ HMGA1 enhances self-renewal and expands the Paneth cell niche by amplifying Wnt signaling.^48^ The expression of HMGA2 decreases as stem cells age.^49^ HMGA2 promotes the self-renewal of neural stem cell and inhibits the expression of genes associated with senescence, specifically *p16*^*Ink4a*^ and *p19*^*Arf*^.^50^ Elucidating the signaling pathways involved in NUP62-regulated neurodegeneration, as well as how NUP62 promotes the rejuvenation of neural stem cells, will represent a significant conceptual advancement. In our study, NUP62 may enhance the renewal and differentiation potential of HDPSCs through the epigenetic regulation of HMGA1 and HMGA2 expression. Additionally, SIRT6, a well-known longevity gene, plays a crucial role in aging-related diseases.^51,52^ Increased levels of SIRT6 in vivo are associated with a longer lifespan.^52,53^ Consequently, NUP62 may ameliorate the senescence-associated phenotypes of HDPSCs by enhancing DNA damage repair through the upregulation of SIRT6.

HDPSCs possess proliferative, multilineage differentiation and immunomodulatory abilities.^7,9^ Numerous studies have showed their potential for treating age-related diseases, such as osteoarthritis, Parkinson’s disease, and Alzheimer’s disease.^8,54^ However, HDPSCs exhibit a decline in both proliferative capacity and regenerative efficiency as age increases, which involve different molecular mechanisms.^10,11^ Targeting NUP62 could enhance the regenerative efficiency of O-HDPSCs, potentially supporting the clinical application of HDPSCs in regenerative medicine. Currently, there is no evidence to suggest that NUP62 improves the regenerative capacity of HDPSCs by alleviating senescence. Therefore, it remains to be explored whether NUP62 directly regulates the differentiation of HDPSCs through histone modification of differentiation-related genes. Additionally, further study is needed to understand the role of NUP62 in other types of MSCs.

In summary, our studies revealed that NUP62 plays a prominent role in the senescence of HDPSC by regulating the expression of NSD2. This regulation involves regulating the nuclear transport of the transcription factor E2F1, which outlines a series of molecular events that remodel chromatin. Our study revealed the key mechanism by which NUP62 operates in the senescence of HDPSC. This discovery paves the way for future studies that may extend to other Nups or to other adult stem cells, enhancing our understanding of how Nups are involved in chromatin remodeling and aging-related diseases.

## Materials and Medthods

### HDPSC isolation and culture

The dental pulp was obtained from surgically extracted intact human third molars from patients aged 16 to 70 years. Based on findings from previous literatures^12,55,56^ and proteomic clustering from our earlier study,^57^ participants were categorized into three groups: young (<23 years), middle-aged (23–40 years), and old (>40 years). The study was approved by the Biomedical Ethics Committee of Ninth People’s Hospital, Shanghai Jiao Tong University School of Medicine (No. SH9H‒2022‒TK510‒1), and informed consent forms were obtained from the participants or their guardians.

The teeth were rinsed with sterile phosphate-buffered saline (PBS) before isolating pulp tissue. The collected pulp tissue was minced into pieces and digested with 4 mg/mL dispase (Gibco, Grand Island, NY, USA) and 3 mg/mL collagenase type I (Invitrogen Life Technology, Carlsbad, CA, USA) at 37 °C for 1 h. Then, the digested single cells were collected and plated into a 10 cm culture dish with α-modified minimum essential medium (α-MEM) supplemented with 1% penicillin-streptomycin (Beyotime, China) and 20% fetal bovine serum (FBS, Biolnd, Israel). The dishes were incubated at 37 °C in 5% CO_2_.

### Flow cytometry

Isolated HDPSCs were analyzed for surface marker expression using flow cytometry. HDPSCs at passage 3 were collected through trypsin-EDTA digestion and resuspended in PBS. A total of 5 × 10^5^ cells dispersed in 600 μL of PBS were transferred to test tubes, incubated with immunophenotype antibodies (Table S1) for 30 min, and then analyzed using a flow cytometer (BD, LSR Fortessa X‒20). The data were further analyzed with FlowJo (version 10.4) software.

### Lentiviral transfection of HDPSCs

NUP62 overexpression was induced in HDPSCs using adenoviral NUP62 constructs at a multiplicity of infection (MOI) of 10, with empty vector (Vector) used as a control, in the presence of polybrene (Hanheng, 8 ng/mL). Stable cells were generated selecting with puromycin (Geman, 2 μg/mL). The efficiency of NUP62 overexpression was assessed using Western blotting.

### Knockdown of NUP62

HDPSCs were infected with a lentiviral vector containing control or NUP62-targeting short hairpin RNA (shRNA) at MOI of 20. Following infection, the cells were selected with puromycin (Geman, 2 μg/mL). The efficiency of NUP62 depletion was assessed using Western blotting. The specific shRNA sequences are listed in Table S2.

### siRNA transfection

Small interfering RNAs (siRNAs) targeting NSD2 or E2F1 were purchased from RiboBio Co., Ltd (Guangzhou, China) and transfected into HDPSCs at a concentration of 25 nmol/L with JetPrimer (No. 101000046, France). The siRNA sequences are listed in Table S2.

### Cell proliferation and migration assay

HDPSCs were seeded in 96-well plates (2 × 10^3^ cells per well). 24 h after seeding HDPSCs into 96-well plates was recorded as Day 0. A Cell Counting Kit-8 (CCK8) (Dojindo, Japan) was used to measure the cell proliferation rate over 7 days. Absorbance was measured at 450 nm using a multiskan (GO, Thermo Scientific). HDPSCs were centrifuged at 1 000 r/min for 5 min to prepare a 1 × 10^5^ cells per mL cell suspension. In the Transwell (Costar, Transwell BD Matrigel, 3422) migration assay, 200 μL of the cell suspension was added into the upper chamber, while 500 μL of culture medium containing FBS was added into the lower chamber. After 24 h of incubation, the culture medium in the upper chamber was replaced with an FBS-free culture medium. Following an additional 24 h of incubation, crystal violet ammonium oxalate solution (Solarbio, China) was added for staining. The cells were then counted and photographed under an inverted microscope (Leica). All the experiments were repeated three times.

### Senescence‐associated beta-galactosidase assay (SA β-gal)

Senescent cells were detected using a β-gal staining kit (Solarbio, China). The culture medium was removed from the six-well plates, which were then washed once with PBS. The plates were fixed for 15 min with 1 mL of β-gal staining fixative buffer. After fixing, the cells were washed with PBS 3 times, and 1 mL of staining solution was added to each well. Six well plates were incubated at 37 °C, and the cells were counted under an ordinary light microscope (DP73, Olympus).

### Differentiation Induction

HDPSCs were cultured in osteogenic differentiation medium containing 1% penicillin‒streptomycin, 10% FBS, 0.2% ascorbate, 1% glutamine, 1% β-glycerophosphate, and 0.01% dexamethasone.

HDPSCs were grown in an adipogenic medium consisting of 10% FBS, 100 μg/mL isobutyl-methylxanthine, 1 μmol/L dexamethasone, and 10 μg/mL insulin. After 28 days of cultivation, lipid droplet formation was detected using Oil Red O staining (Cyagen, USA).

For chondrogenic differentiation, HDPSCs were grown in a medium consisting of 10% FBS, 2 ng/mL transforming growth factor-β (TGF-1β), 50 μmol/L ascorbic acid-2-phosphate, and 10 nmol/L dexamethasone. After 21 days of cultivation, the cell pellets were fixed and cut into 5 μm thick paraffin sections. Alcian blue staining (Cyagen, USA) was performed to assess the deposition of glycosaminoglycans.

Additionally, HDPSCs were grown in neurobasal Medium (Gibco, Life Technologies, Carlsbad, CA) consisting of 1% B27 (Gibco, Life Technologies), 20 ng/mL epidermal growth factor (Thermo), 1% penicillin-streptomycin, and 40 ng/mL fibroblast growth factor 2 (Thermo).

### Alizarin red staining and mineralization assay

After 21 days of osteogenic differentiation induction, the cells were fixed with 4% paraformaldehyde and stained with 1% Alizarin Red S solution for 10 min (Cyagen, USA). The mineralized matrix was destained with 10% cetylpyridinium chloride for 30 min. The calcium concentration was evaluated by determining the optical density at 562 nm with a multiscan (GO, Thermo Scientific).

### Immunofluorescence staining

A cell suspension at a concentration of 2 × 10^4^ cells per mL was prepared and then seeded into 24-well plates loaded with 15 mm glass slides. The plates were incubated at 37 °C for 24 h. Following this, the cells were washed with PBS, fixed in 4% paraformaldehyde for 20 min, and then treated with 0.5% Triton X-100 (Sigma‒Aldrich, #SLCF3053) to enhance cell membrane penetrability. After blocking in 5% BSA for 30 min, the cells were incubated overnight at 4 °C with primary antibodies against NUP62 (Abcam, ab96134, 1:200), γH2AX (Cell Signaling Technology, #9718T, 1:200), H3K36me2 (Active Motif, 39056, 1:200), H3K36me3 (Cell Signaling Technology, #4909S, 1:200) or E2F1 (Cohesion, CQA8351, 1:100). The next day, the cells were incubated for 1 h at 37 °C with fluorescently labeled secondary antibodies (Invitrogen, 1:200). Nuclei were stained with DAPI (Sigma-Aldrich, 1:1 000) for 10 min. Images were collected with a microscope (DP73, Olympus), and further analyses was performed using ImageJ software. To quantify immunofluorescence microscopy images, five random fields images were used to determine intensity of NUP62, γH2AX, H3K36me2 and H3K36me3 per cell. To quantify the Nuclear/Cytoplasmic fluorescence ratio of E2F1, images were used to determine the distribution of E2F1 in the nucleus and cytoplasm per cell (*n* = 10). To ensure reliable quantification, all samples in the same experiment were imaged under the same scanning conditions.

### Nuclear cytoplasmic protein fractionation

Nuclear cytoplasmic protein fractionation was performed using NE-PER® Nuclear and Cytoplasm Extraction Reagents (Thermo Scientific, #78833).

### Western blotting

HDPSCs were lysed in cell lysis buffer (Cell Signaling Technology, #9803) supplemented with 1 mmol/L phenylmethylsufonyl fluoride (Beyotime, China) and protease inhibitor cocktail (Millipore, #539134). Denatured proteins (20 μg) were subjected to SDS‒polyacrylamide gel electrophoresis and then transferred on to a polyvinylidene difluoride (Millipore, USA) membrane at 80 V for 1.5 h. After blocking for 2 h in 5% nonfat milk dissolved in Tris-buffered saline Tween 20 (TBST), the membranes were incubated with primary antibodies against P21 (Cell Signaling Technology, #2947S, 1:1 000), GAPDH (Abcam, ab8245, 1:5 000), NUP62 (Abcam, ab140651, 1:1 000), P53 (Santa Cruz, sc126, 1:500), RUNX2 (Cell Signaling Technology, #12556, 1:1 000), Osterix (Invitrogen, PA5-40509, 1:1 000), DSPP (Santa Cruz, sc73632, 1:500), DMP1 (Invitrogen, PA5-88069, 1:1 000), SOX2 (Cell Signaling Technology, #3579S, 1:1 000), GFAP (Abcam, ab207165, 1:1 000), MAP2 (Affinity, AF4081, 1:1 000), H3 (Cell Signaling Technology, #4499T, 1:1 000), H3K36me2 (Active Motif, 39056, 1:1 000), H3K36me3 (Cell Signaling Technology, #4909S, 1:1 000), H3K9me3 (Abcam, ab75359, 1:1000), H3K27ac (Active Motif, 39085, 1:1 000), H3K4me3 (Cell Signaling Technology, 9751 T, 1:1 000), NSD2 (Abcam, ab75359, 5 μg/mL), E2F1 (Cell Signaling Technology, #3742S, 1:1 000) or β-tubulin (Huaxingbio, HX1984, 1:5 000) overnight at 4 °C. The membranes were washed with TBST 3 times and then incubated with goat-anti-rabbit or goat-anti-mouse secondary antibodies for 2 h. After the membranes were washed 3 times with TBST, they were visualized using a chemiluminescence imaging system (ChemiDoc Systern, BioRad).

### Real-time polymerase chain reaction

Total RNA was extracted from HDPSCs using TRIzol (Takara). A SuperScript Reverse Transcriptase Kit (Takara) was utilized to synthesize complementary DNA (cDNA) for analysis of mRNA expression. Quantitative PCR (qPCR) was performed with a Light Cycler (Roche) using specific primers (Table S3). The relative gene expression levels were analyzed using the 2^-∆∆CT^ method and normalized to that of GAPDH.

### Luciferase reporter assay

293 T cells were obtained from the Chinese Academy of Sciences Cell Bank (Shanghai, China). These cells were seeded into 24-well plates (5 × 10^4^ cells per well). Once the cells reached 70% to 80% confluence, they were transfected with JetPrimer (No. 101000046, France). The transfection mixture contained 160 ng of pGL3 basic-promoter, 10 ng of pRL-Renilla, and 160 ng of either an E2F1 expression vector or red fluorescent protein control. Luciferase activity was measured with a dual-luciferase assay kit (Yeasen, 11405ES60) 48 h after transfection. Specifically, 80 μL of lysate was mixed with 80 μL of luciferase buffer, and the luciferase activity was analyzed with an automatic luminometer (Tecan Infinite Lumi). Then, 80 μL of Stop & Glo reagent was added, and Renilla luminescence was measured after 10 min of incubation. The ratio of Firefly to Renilla luciferase activity was calculated to determine promoter activity. The promoter region sequences of NSD2 were provided in Table S4.

### Transcription profiling of NUP62-overexpressing HDPSCs

We sequenced the RNA from HDPSCs 48 h after transfection with adenoviral constructs containing an empty vector or those overexpressing NUP62 (*n* = 9). RNA was extracted using the RNeasy Plus Kit from QIAGEN. Total RNA samples were subjected to poly (A)-selected sequencing library preparation with the TruSeq RNA Sample Prep Kit version 2 (Illumina). The abundance of library scripts (fragments per kilobase of exons per million mapped reads) was calculated using TopHat and Cufflinks software in conjunction with the human reference genome (hg19). GSEA software (version 4.3.2) was used for GSEA analysis.

### Cleavage Under Targets and Tagmentation (CUT&Tag) Sequencing

HDPSCs were transfected with adenoviral constructs containing either an empty vector or overexpressing NUP62. The cells were then counted, harvested, and centrifuged for 5 min at 300 × *g*. Following this, they were washed twice with wash buffer. Concanavalin A magnetic-coated beads (Vazyme) were activated by washing twice in binding buffer. The beads were separated using a magnet, and the supernatant was removed. Five microliters of H3K36me2 (Active Motif, 39056) antibody or 1 μL of H3K36me3 (Cell Signaling Technology, #4909S) antibody was added to 200 μL of antibody buffer and incubated overnight at 4 °C. The Hyperactive In-Situ ChIP Library Pre Kit for Illumina (pG-Tn5) (Vazyme) was used for CUT&Tag. The data were visualized using IGV (version 2.16.2) along with the human reference genome (hg19).

### Chromatin immunoprecipitation

Chromatin from crosslinked HDPSCs transfected with adenoviral constructs containing either an empty vector or overexpressing NUP62 was sonicated, precleared, and incubated overnight with antibodies in RIPA buffer. This mixture was then precipitated with protein G magnetic beads (Cell Signaling Technology, #9006) for 2 h. The antibodies used for the ChIP assay were H3K36me2 (Active Motif, 39056, 3 μg per test), H3K36me3 (Cell Signaling Technology, #4909S, 3 μg per test), NSD2 (Millipore Sigma, MABE191, 4 μg per test), E2F1 (Cell Signaling Technology, #3742S, 4 μg per test) and IgG (Cell Signaling Technology, #3900S, 4 μg per test). The DNA-protein-antibody complexes were washed once with Low Salt Wash Buffer (LS), once with High Salt Wash Buffer (HS), once with LiCl Wash Buffer, and twice with TE Buffer. The cross-linking of the co-precipitated DNA-protein complexes was reversed with Dr. GenTLE^TM^ Precipitation Carrier (TaKaRa, #9094). The immunoprecipitated DNA was then analyzed by RT‒PCR with primers listed in Table S5. The data are presented as the percentage of input DNA or the fold enrichment of the promoter (target/IgG).

### Rat calvarial bone defects were reconstructed by HDPSC scaffolds

Six- to eight-week-old male Sprague-Dawley rats were used to evaluate bone regeneration in vivo. Ethical approval was received from Shanghai Ninth People’s Hospital (No. SH9H‒2022‒TK510‒1). After exposing the calvarial bone, a critical-size defect was created with an 8 mm dental trephine. A total of 5 × 10^5^ HDPSCs were seeded into a commercialized collagen sponge (6 mm × 6 mm × 3 mm, Helistat 1690ZZ) and inserted into each defect. After 8 weeks, the animals were euthanized with CO_2_, and the repaired skulls were removed. Micro‒CT (Skyscan1176, USA Bruker) was used for the examination of calvarial bone defect repair. Images were 3D reconstructed with ctvox (Bruker micro‒CT, Kontich, Belgium). Bone mineral density (g/cc) was measured and analyzed. Sections (5 μm) were prepared using a microtome for hematoxylin-eosin (H&E) staining.

### Tumorigenic capacity

Female BALB/c nude mice (4 weeks old) were used to evaluate the tumorigenic capacity in vivo. Ethical approval for the animal experiments was received from Shanghai Ninth People’s Hospital (No. SH9H‒2022‒TK510‒1). A total 5 × 10^5^ Y-HDPSCs or O-HDPSCs transfected with an empty vector or overexpression of NUP62 were seeded into a collagen sponge (3 mm×3 mm×3 mm, Helistat 1690ZZ) and subsequently subcutaneously transplanted into both flanks of the nude mice (*n* = 6). After 6 weeks, the animals were euthanized with CO_2_. The transplants were surgically removed, fixed in 4% paraformaldehyde, and prepared for histological analysis.

### Immunohistochemical analysis

Sections (5 μm) of calvarial bone were prepared with a microtome for immunohistochemical staining. After deparaffinization and rehydration, the slices were processed according to the manufacturer’s protocols. The anti-Coll1A1 antibody and anti-OCN antibody were purchased from Santa Cruz and Servicebio. Images were collected with a microscope (Leica, Germany).

### Proteomic analysis and bioinformatic analysis

Proteomic analysis of HDPSC samples was conducted at the Institute of Human Phenome, Fudan University, Shanghai, China. The raw mass spectrometry files were processed using “Firmiana” (a one-stop proteomic cloud platform) against the human National Center for Biotechnology Information (NCBI) RefSeq protein database (updated on April 7, 2013; 32 015 entries).

### Statistical Methods

All quantitative data are shown as the mean ± standard deviation (SD). Analyses and graphical presentations were performed with the GraphPad Prism 8 software. One-way ANOVA analysis was used to indicate differences among multiple groups, while two-tailed Student’s *t* tests were used for comparisons between two independent groups. All results represent two or more independent repeats. The following *p*-value indication scheme was used: ns: *P* > 0.05, *P < 0.05; **P < 0.01; ***P < 0.001.

## Supplementary information

Supplemental material

## Data Availability

The data used and/or analyzed during the current study are contained within the manuscript or available to editors and reviewers prior to publication. All the data will be published at the same time as the article.

## References

[1] Crimmins, E. M. Lifespan and Healthspan: Past, Present, and Promise. *Gerontologist* **55**, 901–911 (2015). 26561272 10.1093/geront/gnv130 PMC4861644

[2] Guo, J. et al. Aging and aging-related diseases: from molecular mechanisms to interventions and treatments. *Signal Transduct. Target Ther.* **7**, 391 (2022). 36522308 10.1038/s41392-022-01251-0 PMC9755275

[3] López-Otín, C., Blasco, M. A., Partridge, L., Serrano, M. & Kroemer, G. The hallmarks of aging. *Cell* **153**, 1194–1217 (2013). 23746838 10.1016/j.cell.2013.05.039 PMC3836174

[4] Maria et al. Cellular and epigenetic drivers of stem cell ageing. *Nat. Rev.: Mol. Cell Biol.* **19**, 594–610 (2018). 29858605 10.1038/s41580-018-0020-3

[5] Ibrayeva, A. et al. Early stem cell aging in the mature brain. *Cell Stem Cell* **28**, 955–966 (2019). 10.1016/j.stem.2021.03.018 PMC10069280 33848469

[6] Gronthos, S., Mankani, M., Brahim, J., Robey, P. G. & Shi, S. Postnatal human dental pulp stem cells (DPSCs) in vitro and in vivo. *Proc. Natl Acad. Sci. USA* **97**, 13625–13630 (2000). 11087820 10.1073/pnas.240309797 PMC17626

[7] Kaukua, N. et al. Glial origin of mesenchymal stem cells in a tooth model system. *Nature* **513**, 551–554 (2014). 25079316 10.1038/nature13536

[8] Achilleos, A. & Trainor, P. A. Neural crest stem cells: discovery, properties and potential for therapy. *Cell Res.* **22**, 288–304 (2012). 22231630 10.1038/cr.2012.11 PMC3271580

[9] Sui, B. et al. Dental pulp stem cells: from discovery to clinical application. *J. Endod.* **46**, S46–S55 (2020). 32950195 10.1016/j.joen.2020.06.027

[10] Yang, R. L. et al. Serine metabolism controls dental pulp stem cell aging by regulating the DNA methylation of p16. *J. Dent. Res.* **100**, 90–97 (2021). 32940141 10.1177/0022034520958374

[11] Iezzi, I., Cerqueni, G., Licini, C., Lucarini, G. & Mattioli Belmonte, M. Dental pulp stem cells senescence and regenerative potential relationship. *J. Cell Physiol.* **234**, 7186–7197 (2019). 30362542 10.1002/jcp.27472

[12] Feng, X. et al. p16(INK4A) mediates age-related changes in mesenchymal stem cells derived from human dental pulp through the DNA damage and stress response. *Mech. Ageing Dev.* **141-142**, 46–55 (2014). 25304494 10.1016/j.mad.2014.09.004

[13] Dong, X. Y. et al. Downregulation of ROR2 promotes dental pulp stem cell senescence by inhibiting STK4-FOXO1/SMS1 axis in sphingomyelin biosynthesis. *Aging Cell* **20**, e13430 (2021). 34278704 10.1111/acel.13430 PMC8373368

[14] Luo, H., Liu, W., Zhang, Y., Yang, Y. & Shao, L. METTL3-mediated mA modification regulates cell cycle progression of dental pulp stem cells. *Stem Cell Res. Ther.* **12**, 159 (2021). 33648590 10.1186/s13287-021-02223-x PMC7923612

[15] Patel, S. S., Belmont, B. J., Sante, J. M. & Rexach, M. F. Natively unfolded nucleoporins gate protein diffusion across the nuclear pore complex. *Cell* **129**, 83–96 (2007). 17418788 10.1016/j.cell.2007.01.044

[16] D’Angelo, M A. & Raices, M. Nuclear pore complexes and regulation of gene expression. *Curr. Opin. Cell Biol.* **46**, 26–32 (2017). 28088069 10.1016/j.ceb.2016.12.006 PMC5505778

[17] Beck, M. & Hurt, E. The nuclear pore complex: understanding its function through structural insight. *Nat. Rev. Mol. Cell Biol.* **18**, 73–89 (2017). 27999437 10.1038/nrm.2016.147

[18] Breuer, M. & Ohkura, H. A negative loop within the nuclear pore complex controls global chromatin organization. *Genes Dev.* **29**, 1789–1794 (2015). 26341556 10.1101/gad.264341.115 PMC4573852

[19] Liu, J. & Hetzer, M. W. Nuclear pore complex maintenance and implications for age-related diseases. *Trends Cell Biol.* **32**, 216–227 (2022). 34782239 10.1016/j.tcb.2021.10.001

[20] Cho, U. H. & Hetzer, M. W. Nuclear periphery takes center stage: the role of nuclear pore complexes in cell identity and aging. *Neuron* **106**, 899–911 (2020). 32553207 10.1016/j.neuron.2020.05.031 PMC9041311

[21] Coyne, A. N. & Rothstein, J. D. Nuclear pore complexes - a doorway to neural injury in neurodegeneration. *Nat. Rev. Neurol.* **18**, 348–362 (2022). 35488039 10.1038/s41582-022-00653-6 PMC10015220

[22] Sun, J., Shi, Y. & Yildirim, E. The nuclear pore complex in cell type-specific chromatin structure and gene regulation. *Trends Genet.* **35**, 579–588 (2019). 31213386 10.1016/j.tig.2019.05.006

[23] Grima et al. Mutant Huntingtin disrupts the nuclear pore complex. *Neuron* **94**, 93–107 (2017). 28384479 10.1016/j.neuron.2017.03.023 PMC5595097

[24] Lee, B. Y. et al. Senescence-associated beta-galactosidase is lysosomal beta-galactosidase. *Aging Cell* **5**, 187–195 (2006). 16626397 10.1111/j.1474-9726.2006.00199.x

[25] Sun, J. et al. A Glb1-2A-mCherry reporter monitors systemic aging and predicts lifespan in middle-aged mice. *Nat. Commun.* **13**, 7028 (2022). 36396643 10.1038/s41467-022-34801-9 PMC9671911

[26] Ertosun, M. G., Hapil, F. Z. & Osman Nidai, O. E2F1 transcription factor and its impact on growth factor and cytokine signaling. *Cytokine Growth Factor Rev.* **31**, 17–25 (2016). 26947516 10.1016/j.cytogfr.2016.02.001

[27] Chun, J. N., Cho, M., Park, S., So, I. & Jeon, J. H. The conflicting role of E2F1 in prostate cancer: A matter of cell context or interpretational flexibility? *Biochim Biophys. Acta Rev. Cancer* **1873**, 188336 (2020). 31870703 10.1016/j.bbcan.2019.188336

[28] Hazawa, M. et al. ROCK-dependent phosphorylation of NUP62 regulates p63 nuclear transport and squamous cell carcinoma proliferation. *EMBO Rep.* **19**, 73–88 (2018). 29217659 10.15252/embr.201744523 PMC5757218

[29] Ocampo, A. et al. InVivo amelioration of age-associated hallmarks by partial reprogramming. *Cell* **167**, 1719–1733 (2016). 27984723 10.1016/j.cell.2016.11.052 PMC5679279

[30] Rando, T. A. & Chang, H. Y. Aging, rejuvenation, and epigenetic reprogramming: resetting the aging clock. *Cell* **148**, 46–57 (2012). 22265401 10.1016/j.cell.2012.01.003 PMC3336960

[31] Gleixner, A. M. et al. NUP62 localizes to ALS/FTLD pathological assemblies and contributes to TDP-43 insolubility. *Nat. Commun.* **13**, 3380 (2022). 35697676 10.1038/s41467-022-31098-6 PMC9192689

[32] Lin, Y.-C. et al. Interactions between ALS-linked FUS and nucleoporins are associated with defects in the nucleocytoplasmic transport pathway. *Nat. Neurosci.* **24**, 1077–1088 (2021). 34059832 10.1038/s41593-021-00859-9 PMC8832378

[33] Borlido, J. & D’Angelo, M. A. Nup62‐mediated nuclear import of p63 in squamous cell carcinoma. *EMBO Rep.* **19**, 3–4 (2018). 29254932 10.15252/embr.201745497 PMC5757215

[34] Li, N. & Lagier-Tourenne, C. Nuclear pores: the gate to neurodegeneration. *Nat. Neurosci.* **21**, 156–158 (2018). 29371653 10.1038/s41593-017-0066-0

[35] Tanaka, H., Igata, T., Etoh, K., Koga, T. & Nakao, M. The NSD2/WHSC1/MMSET methyltransferase prevents cellular senescence-associated epigenomic remodeling. *Aging Cell* **19**, e13173 (2020). 32573059 10.1111/acel.13173 PMC7433007

[36] Nimura, K. et al. A histone H3 lysine 36 trimethyltransferase links Nkx2-5 to Wolf-Hirschhorn syndrome. *Nature* **460**, 287–291 (2009). 19483677 10.1038/nature08086

[37] Zhuang, L. et al. Depletion of Nsd2-mediated histone H3K36 methylation impairs adipose tissue development and function. *Nat. Commun.* **9**, 1796 (2018). 29728617 10.1038/s41467-018-04127-6 PMC5935725

[38] Li, Z. et al. H3K36me2 methyltransferase NSD2 orchestrates epigenetic reprogramming during spermatogenesis. *Nucleic Acids Res.* **50**, 6786–6800 (2022). 35736136 10.1093/nar/gkac533 PMC9262605

[39] Yang, J. H. et al. Loss of epigenetic information as a cause of mammalian aging. *Cell* **186**, 305–326.e327 (2023). 36638792 10.1016/j.cell.2022.12.027 PMC10166133

[40] Pu, M. et al. Trimethylation of Lys36 on H3 restricts gene expression change during aging and impacts life span. *Genes Dev.* **29**, 718–731 (2015). 25838541 10.1101/gad.254144.114 PMC4387714

[41] Payel et al. H3K36 methylation promotes longevity by enhancing transcriptional fidelity. *Genes Dev.* **29**, 1362–1376 (2015). 26159996 10.1101/gad.263707.115 PMC4511212

[42] Fong, N., Saldi, T., Sheridan, R. M., Cortazar, M. A. & Bentley, D. L. RNA Pol II dynamics modulate co-transcriptional chromatin modification, CTD phosphorylation, and transcriptional direction. *Mol. Cell* **66**, 546–557.e543 (2017). 28506463 10.1016/j.molcel.2017.04.016 PMC5488731

[43] Rao, B., Shibata, Y., Strahl, B. D. & Lieb, J. D. Dimethylation of histone H3 at lysine 36 demarcates regulatory and nonregulatory chromatin genome-wide. *Mol. Cell Biol.* **25**, 9447–9459 (2005). 16227595 10.1128/MCB.25.21.9447-9459.2005 PMC1265832

[44] Edmunds, J. W., Mahadevan, L. C. & Clayton, A. L. Dynamic histone H3 methylation during gene induction: HYPB/Setd2 mediates all H3K36 trimethylation. *EMBO J.* **27**, 406–420 (2008). 18157086 10.1038/sj.emboj.7601967 PMC2168397

[45] Wang, L. et al. High Mobility Group A1 (HMGA1): Structure, biological function, and therapeutic potential. *Int J. Biol. Sci.* **18**, 4414–4431 (2022). 35864955 10.7150/ijbs.72952 PMC9295051

[46] Colamaio, M. et al. HMGA1 silencing reduces stemness and temozolomide resistance in glioblastoma stem cells. *Expert Opin. Ther. Targets* **20**, 1169–1179 (2016). 27486901 10.1080/14728222.2016.1220543

[47] De Martino, M. et al. Characterization of HMGA1P6 transgenic mouse embryonic fibroblasts. *Cell Cycle* **19**, 2281–2285 (2020). 32787507 10.1080/15384101.2020.1807080 PMC7513866

[48] Xian, L. et al. HMGA1 amplifies Wnt signalling and expands the intestinal stem cell compartment and Paneth cell niche. *Nat. Commun.* **8**, 15008 (2017). 28452345 10.1038/ncomms15008 PMC5414379

[49] Hammond, S. M. & Sharpless, N. E. HMGA2, microRNAs, and stem cell aging. *Cell* **135**, 1013–1016 (2008). 19070572 10.1016/j.cell.2008.11.026 PMC3725266

[50] Nishino, J., Kim, I., Chada, K. & Morrison, S. J. Hmga2 promotes neural stem cell self-renewal in young but not old mice by reducing p16Ink4a and p19Arf Expression. *Cell* **135**, 227–239 (2008). 18957199 10.1016/j.cell.2008.09.01 PMC2582221

[51] Korotkov, A., Seluanov, A. & Gorbunova, V. Sirtuin 6: linking longevity with genome and epigenome stability. *Trends Cell Biol.* **31**, 994–1006 (2021). 34281779 10.1016/j.tcb.2021.06.009 PMC8903056

[52] Tian, X. et al. SIRT6 Is Responsible for More Efficient DNA Double-Strand Break Repair in Long-Lived Species. *Cell* **177**, 622–638.e622 (2019). 31002797 10.1016/j.cell.2019.03.043 PMC6499390

[53] Roichman, A. et al. Restoration of energy homeostasis by SIRT6 extends healthy lifespan. *Nat. Commun.* **12**, 3208 (2021). 34050173 10.1038/s41467-021-23545-7 PMC8163764

[54] Xiao, Z. et al. The potential therapy with dental tissue-derived mesenchymal stem cells in Parkinson’s disease. *Stem Cell Res Ther.* **12**, 5 (2021). 33407864 10.1186/s13287-020-01957-4 PMC7789713

[55] Balic, A. Biology explaining tooth repair and regeneration: A mini review. *Gerontology* **64**, 5 (2018). 10.1159/000486592 29533942

[56] Bernick, S. & Nedelman, C. Effect of aging on human pulp. *J. Endod.* **1**, 3 (1975). 10.1016/S0099-2399(75)80024-0 1061788

[57] Chen, L. et al. Integrin-linked kinase control dental pulp stem cell senescence via the mTOR signaling pathway. *Stem Cells* **42**, 10 (2024). 10.1093/stmcls/sxae047 PMC11464141 39169713

