# Supplementary material for: NUP62 alleviates senescence and promotes the stemness of human dental pulp stem cells via NSD2-dependent epigenetic reprogramming
Source: Int J Oral Sci. 2025 Apr 17;17:34. doi: 10.1038/s41368-025-00362-y (PMC12006529; doi:10.1038/s41368-025-00362-y)
Supplement: Supplementary file 1 — Supplemental material [file 41368_2025_362_MOESM1_ESM.pdf]

Supplementary Material

NUP62 alleviates senescence and promotes the stemness of human dental pulp stem cells via NSD2-dependent epigenetic reprogramming

Xiping Wang[1, #], Li Wang[1, #], Linxi Zhou[2], Lu Chen[3], Jiayi Shi[1], Jing Ge[3], Sha Tian[4], Zihan Yang[1], Yuqiong Zhou[3], Qihao Yu[1], Jiacheng Jin[5], Chen Ding[4], Yihuai Pan[1,6, *] and Duohong Zou[3, 1, *]

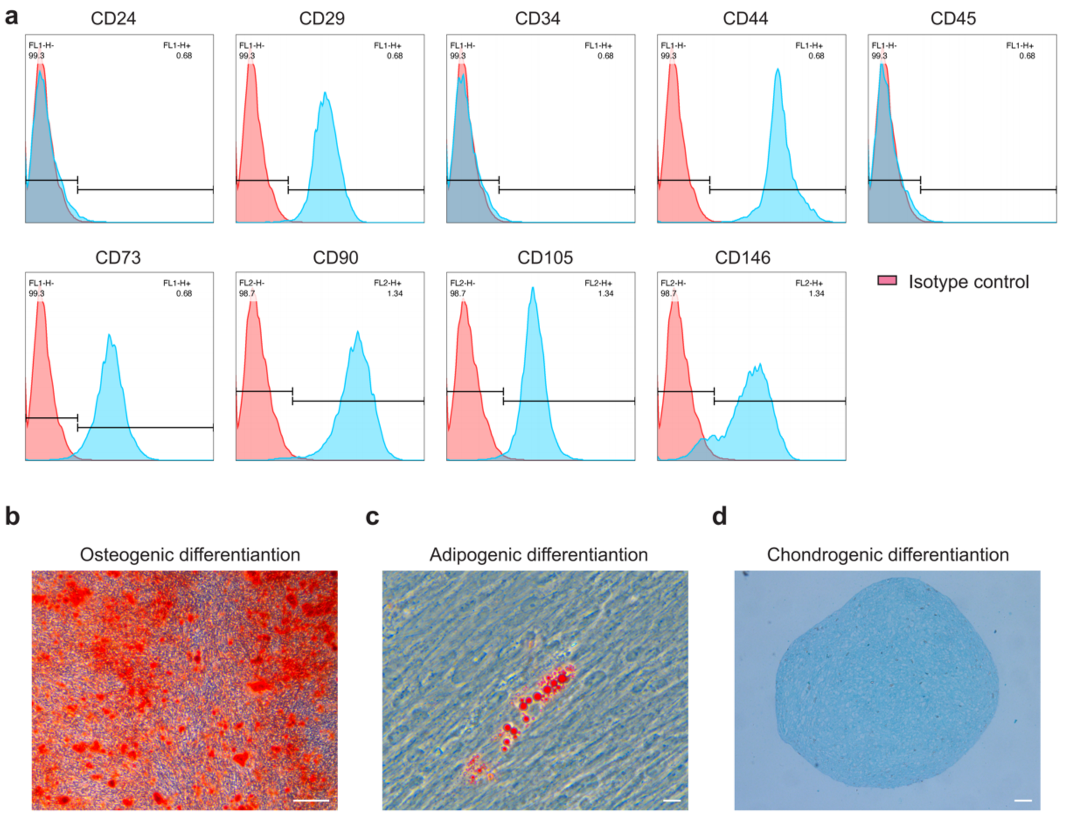

Fig. S1 Immunophenotyping and three-line differentiation of human dental pulp stem cells (HDPSCs). a Representative images of flow cytometric analysis of the surface marker expression. **b** Representative image of Alizarin Red S (ARS) staining

of HDPSCs induced in osteogenic induction medium for up to 21 days. Scale bar, 200 μm. **c** Representative image of Oil red O staining of HDPSCs induced in an adipogenic induction medium for up to 21 days. Scale bar, 20 μm. **d** Representative image of Alcian blue-stained HDPSCs induced in chondrogenic induction medium for up to 21 days. Scale bar, 20 μm.

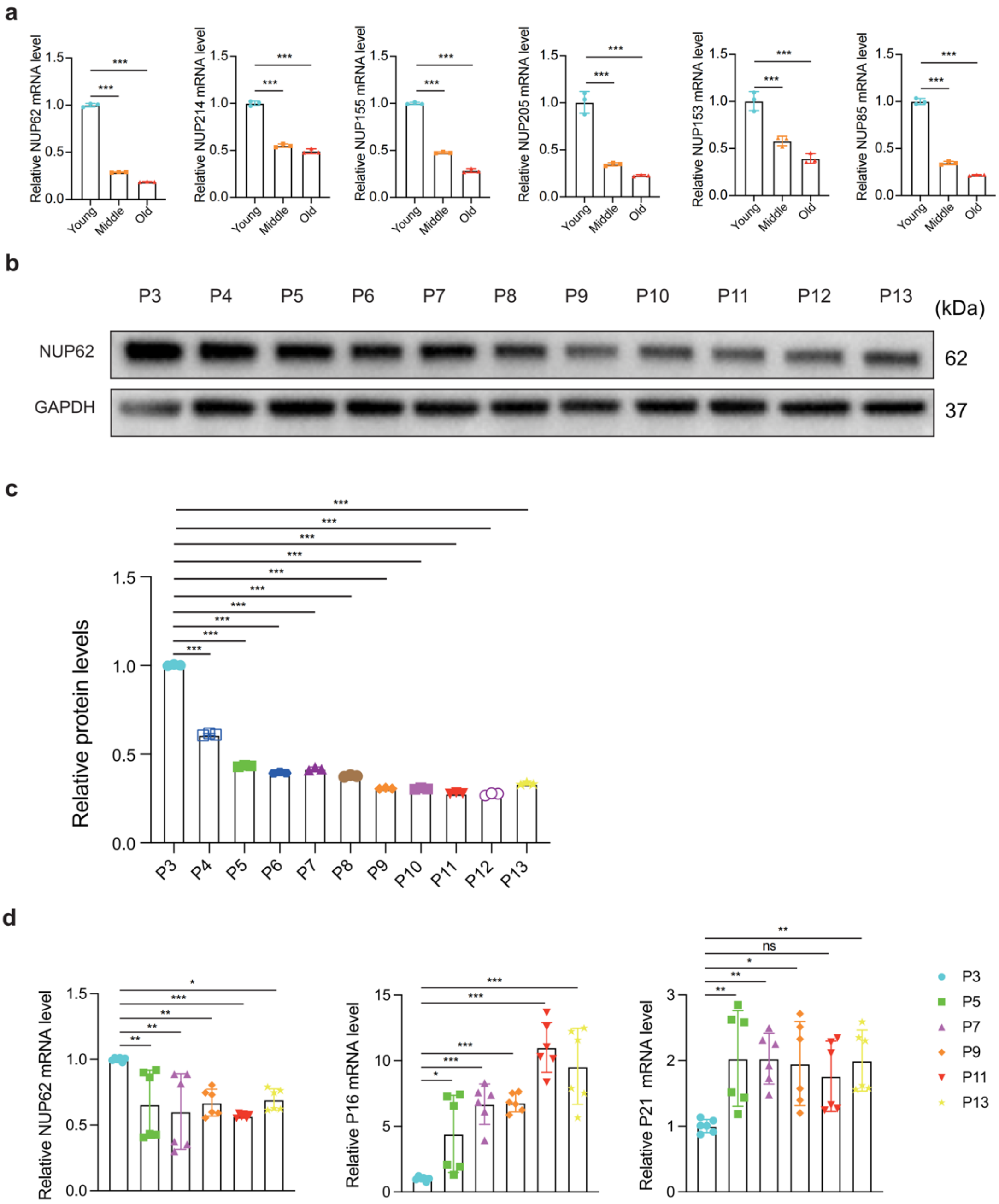

Fig. S2 NUP62 expression decreased in senescent human dental pulp stem cells (HDPSCs). a Quantitative RT–PCR analysis of Nups expression in HDPSCs isolated from different donors (n=3). **b, c** Western blot analysis of NUP62 expression in HDPSCs with increasing passage numbers. **d** Quantitative RT–PCR analysis of the expression of NUP62 and senescence-associated genes (P16 and P21) with increasing HDPSC passage numbers (n=6). **P*<0.05, ***P*<0.01 and ****P*<0.001.

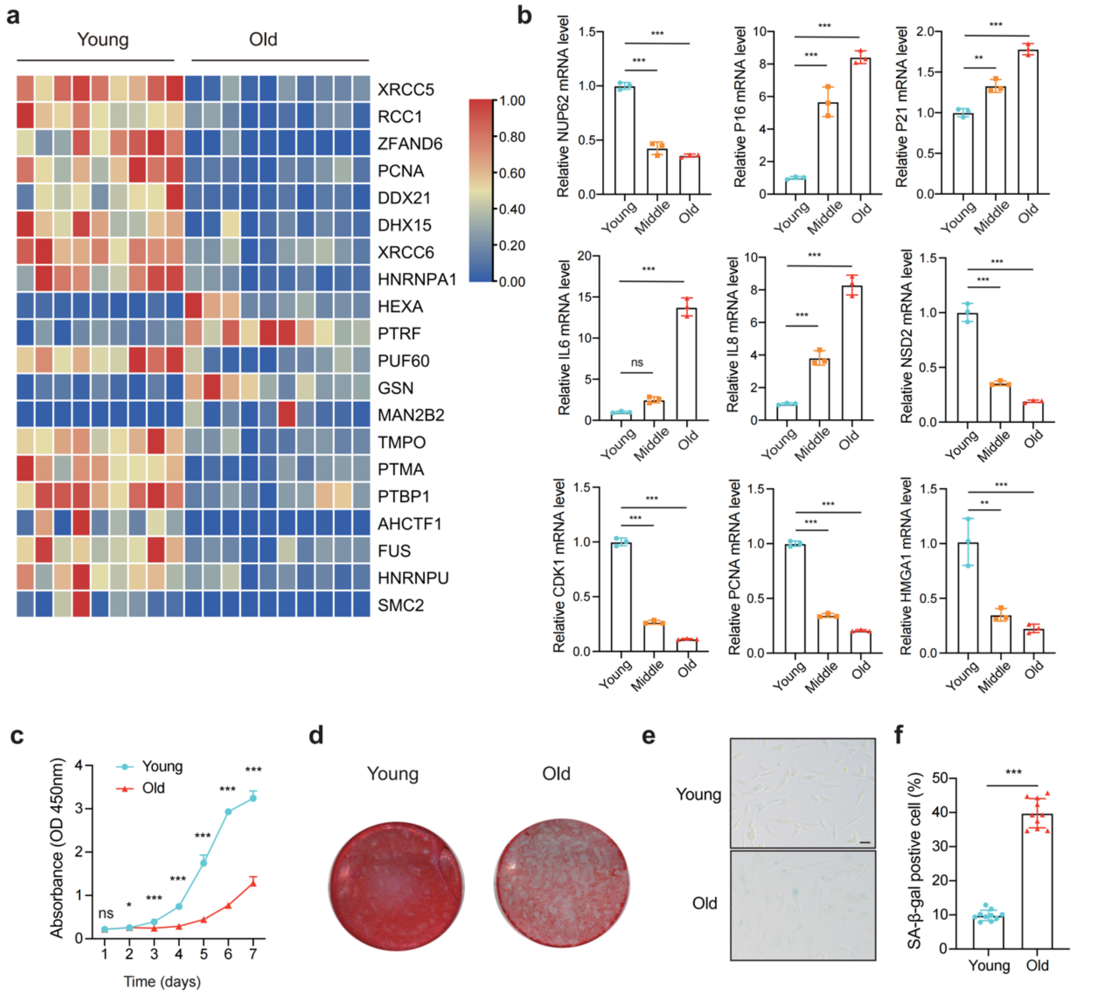

Fig. S3 Human dental pulp stem cells (HDPSCs) showed age-related phenotypes with aging. a. Heatmap of Top 20 proteins that were differentially expressed in HDPSCs isolated from different donors (> 1.5-fold, t test, p < 0.05). **b.** Quantitative

RT–PCR results of NUP62, P16, P21, IL6, IL8, NSD2, CDK1, PCNA and HMGA1 in HDPSCs with age (n=3). **c** CCK-8 assay results (n = 5). **d** Representative images of Alizarin red S staining of HDPSCs induced in an osteogenic induction medium for up to 21 days. **e**, **f** Representative images of senescence-associated β-galactosidase (SA β-gal) staining of HDPSCs and quantification of positive cells (n = 10). Scale bar, 50 μm. **P*<0.05, ***P*<0.01 and ****P*<0.001.

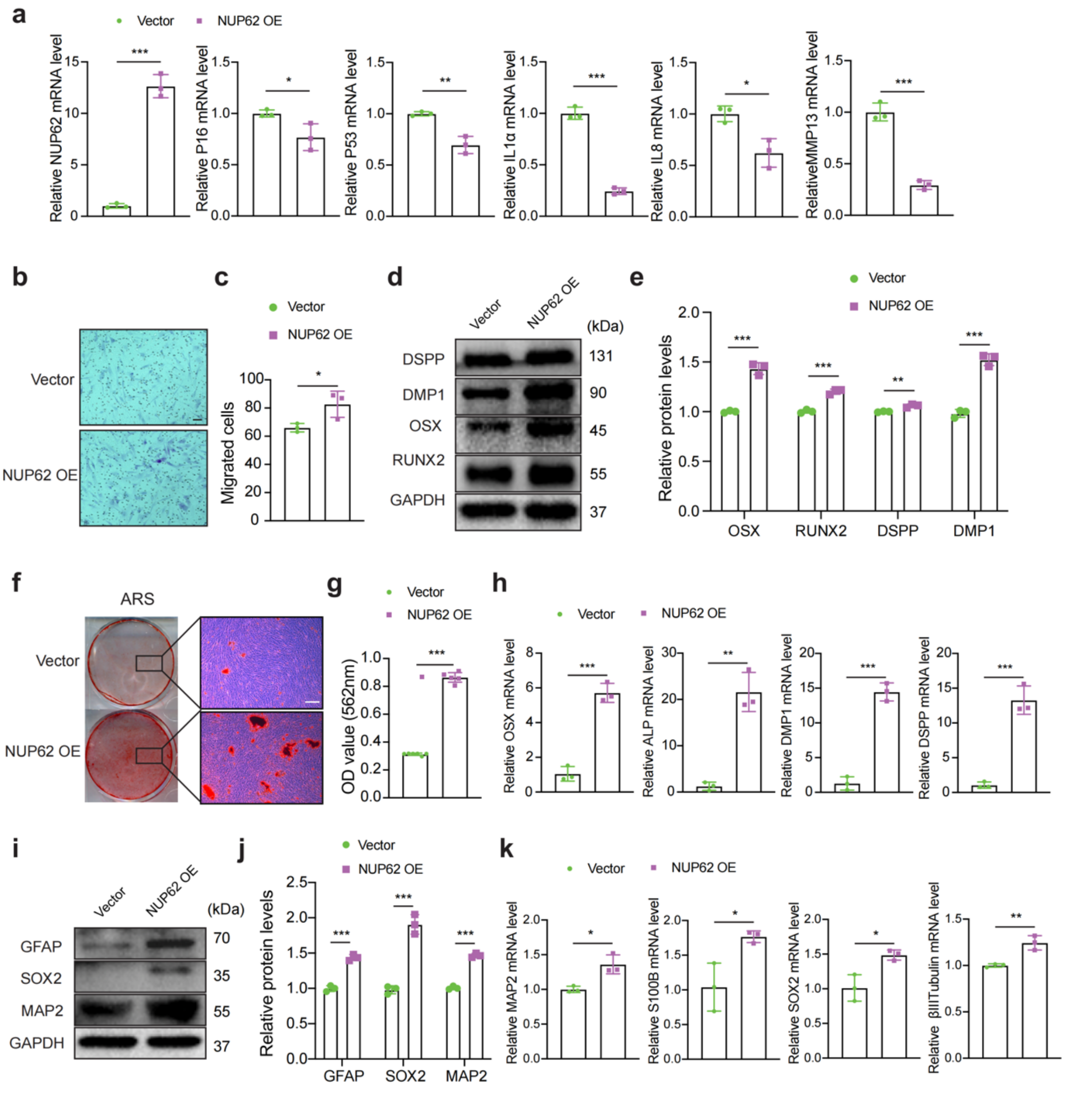

Fig. S4 Overexpression of NUP62 alleviates human dental pulp stem cell (HDPSC) senescence and promotes the differentiation potential of O-HDPSCs. a Quantitative RT–PCR results of NUP62 and senescence-associated genes (P16, P53, IL8, IL1α and MMP13) (n = 3). **b, c** Crystal violet staining of migrated HDPSCs from the upper chamber to the lower bottom of the membrane and quantification of the results (n = 3). Scale bar, 50 μm. **d,e** Western blot analyses of RUNX2, OSX, DMP1, DSPP and GAPDH in lysates of HDPSCs induced in an osteogenic induction medium for up to 14 days. **f** Representative images of Alizarin Red S (ARS) staining of HDPSCs induced in osteogenic induction medium for up to 14 days. Scale bar, 200 μm. **g** Semiquantitative analysis of the ARS signal (n = 6). **h** Quantitative RT–PCR results showing the expression levels of ALP, OSX, DMP1 and DSPP mRNA in HDPSCs induced in an osteogenic induction medium for up to 7 days (n = 3). **i, j** Western blotting for GFAP, SOX2, MAP2 and GAPDH was performed using lysates from HDPSCs induced in a neural induction medium for up to 9 days. **k** Quantitative RT–PCR results showing the expression levels of MAP2, SOX2, S100B and βIII tubulin mRNA in HDPSCs induced in a neural induction medium for up to 7 days (n = 3). *$P<0.05$, **$P<0.01$ and ***$P<0.001$.

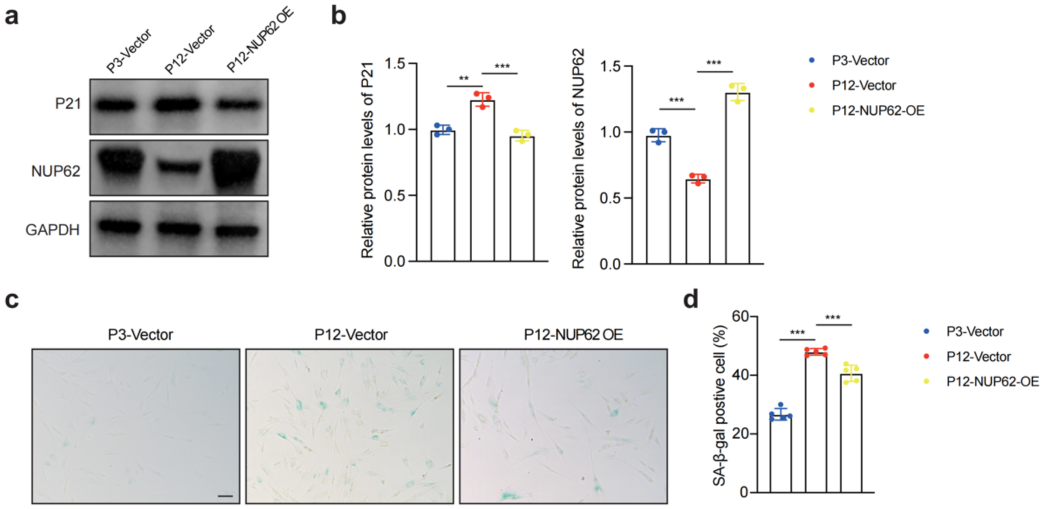

Fig. S5 Overexpression of NUP62 alleviates human dental pulp stem cell (HDPSC) senescence induced by cell passaging. a,b Western blot analyses of NUP62, P21 and GAPDH expression in HDPSCs. **c, d** Representative images of senescence-associated β-galactosidase (SA β-gal) staining of O-HDPSCs and quantification of positive cells (n =5). Scale bar, 50 μm. **P<0.01 and ***P<0.001.

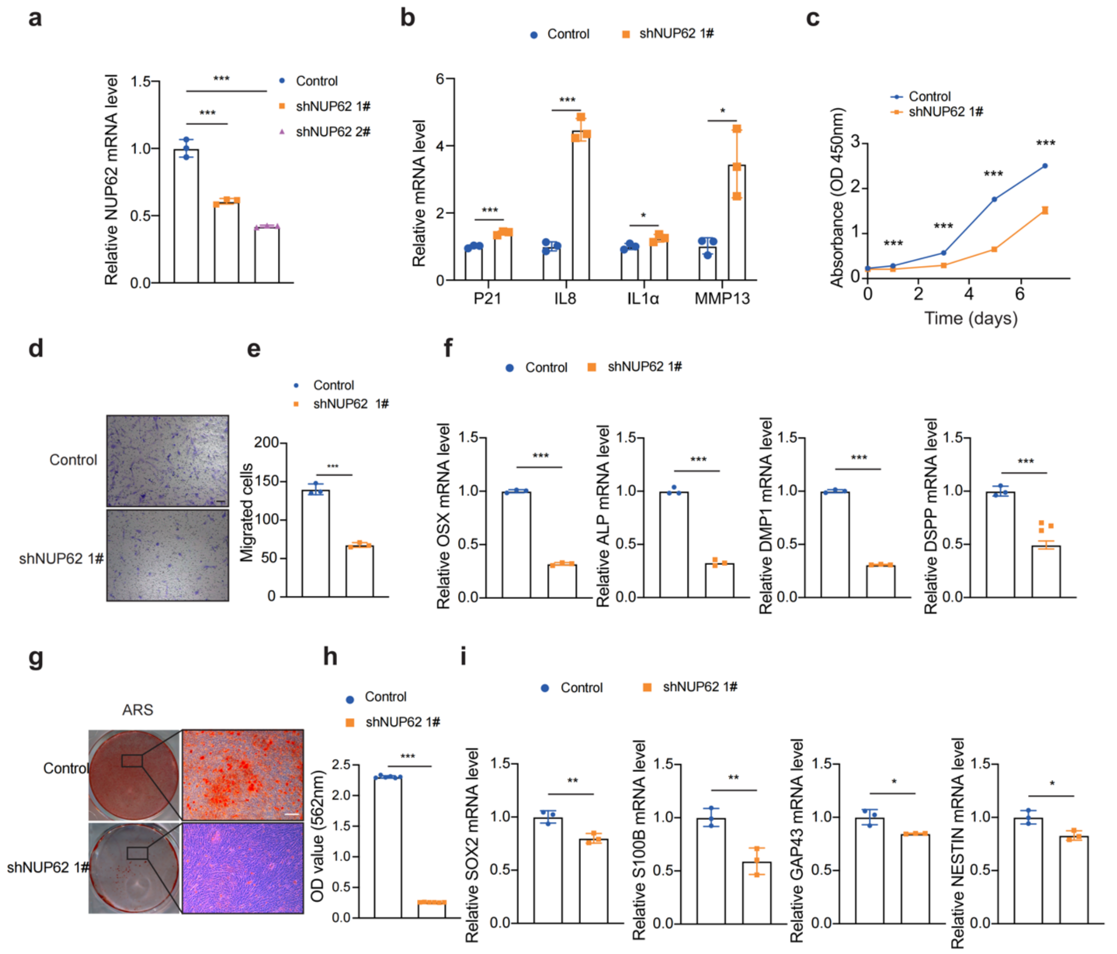

Fig. S6 NUP62 knockdown promotes Y-HDPSC senescence and impaires the differentiation potential of Y-HDPSCs. a Quantitative RT–PCR results showing NUP62 expression (n = 3). **b** Quantitative RT–PCR results showing the expression of senescence-associated genes (P21, IL8, IL1a and MMP13) in HDPSCs (n = 3). **c** CCK-8 assay results (n = 5). **d**, **e** Crystal violet staining of migrated HDPSCs from the upper chamber to the lower bottom of the membrane and quantification of the results (n = 3). Scale bar, 50 μm. **f** Quantitative RT–PCR results showing the mRNA expression levels of OSX, ALP, DMP1 and DSPP in HDPSCs induced in an osteogenic induction medium for up to 7 days (n = 3). **g** Representative images of Alizarin Red S (ARS)

staining of HDPSCs induced in an osteogenic induction medium for up to 14 days. Scale bar, 200 µm. **h** Semiquantitative analysis of the ARS signal (n = 6). **i** Quantitative RT–PCR results showing the expression levels of SOX2, S100B, GAP43 and NSETIN mRNA in HDPSCs induced in a neural induction medium for up to 7 days (n = 3). *P<0.05, **P<0.01 and ***P<0.001.

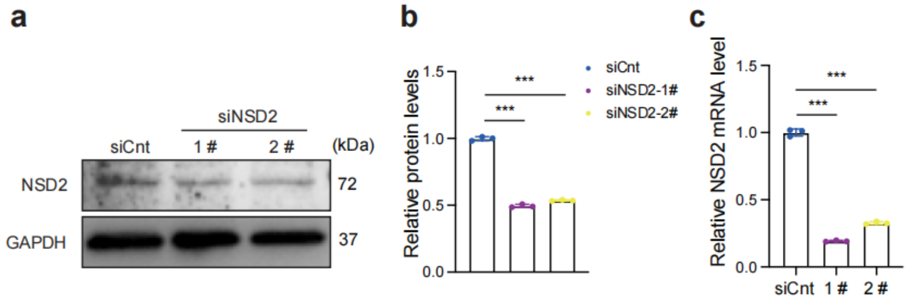

Fig. S7 Knockdown of NSD2 in HDPSCs transfected with siRNA. a, b Western blot analysis of NSD2 in HDPSCs transfected with control siRNA (siCnt) or NSD2 siRNA targeting 2 different sequences (#1, #2). **c** Quantitative RT–PCR analysis of the expression of NSD2 in HDPSCs transfected with control siRNA or NSD2 siRNA (n=3). ***P<0.001.

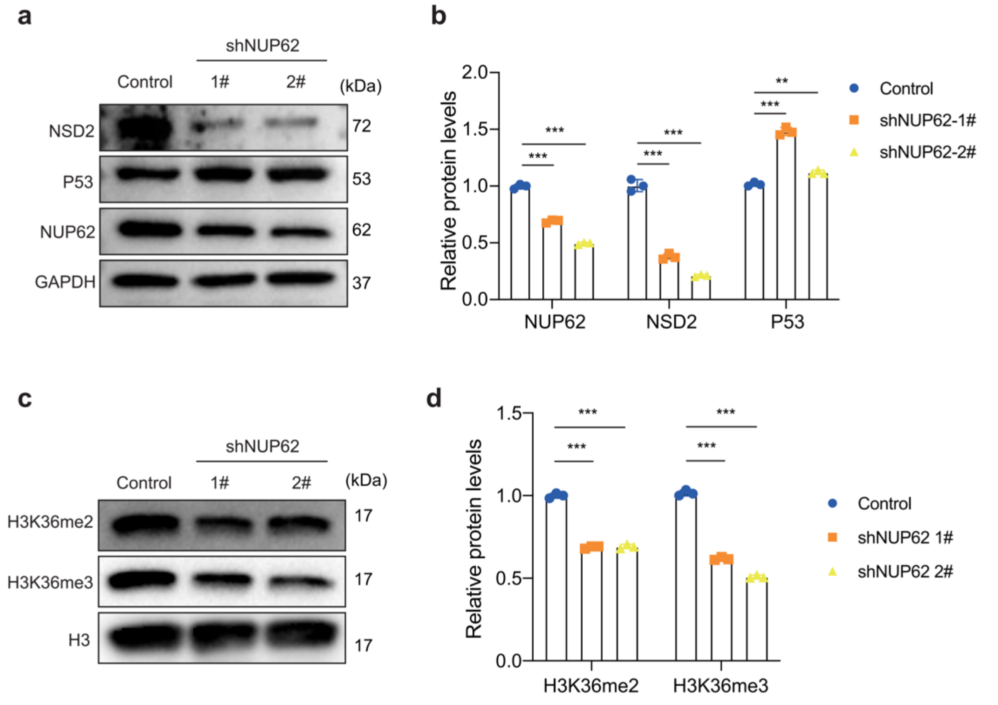

Fig. S8 Knockdown of NUP62 downregulates the expression of NSD2 , H3K36me2, and H3K36me3 levels. a,b Western blot analyses of NSD2, P53, NUP62 and GAPDH expression in HDPSCs with and without NUP62 knockdown. **c,d** Western blot analyses of H3K36me2, H3K36me3 and H3 expression in HDPSCs with and without NUP62 knockdown. ***P*<0.01 and ****P*<0.001.

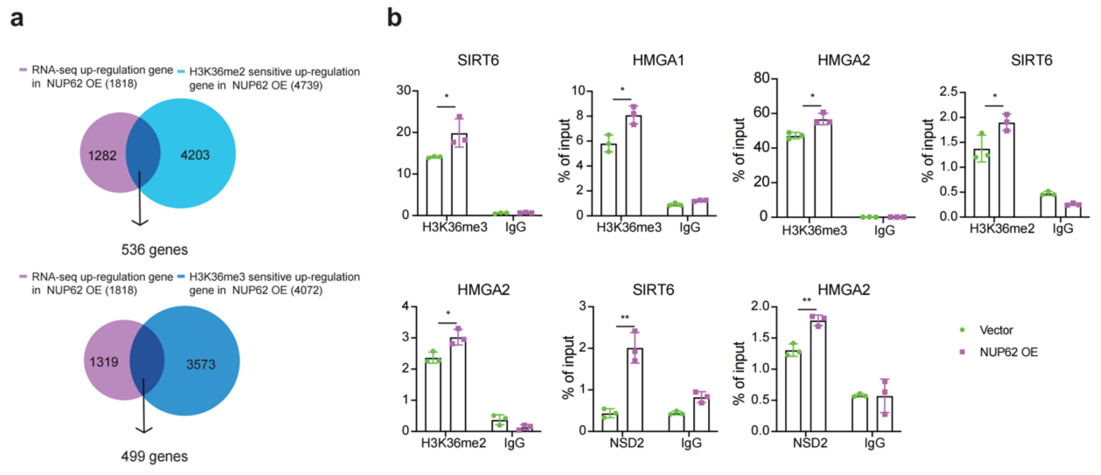

Fig. S9 NUP62-dependent genomic histone modification changes. a Venn diagrams depicting NUP62-dependent upregulated genes (1818) and H3K36me2-sensitive (4739) or H3K36me3-sensitive (4072) upregulated bound peaks at NUP62 overexpression. **b** ChIP–qPCR revealed that overexpression of NUP62 increased the occupancy of H3K36me3, H3K36me2 and NSD2 in the promoter regions of SIRT6, HMGA1 and HMGA2 (n = 3). **P*<0.05 and ***P*<0.01

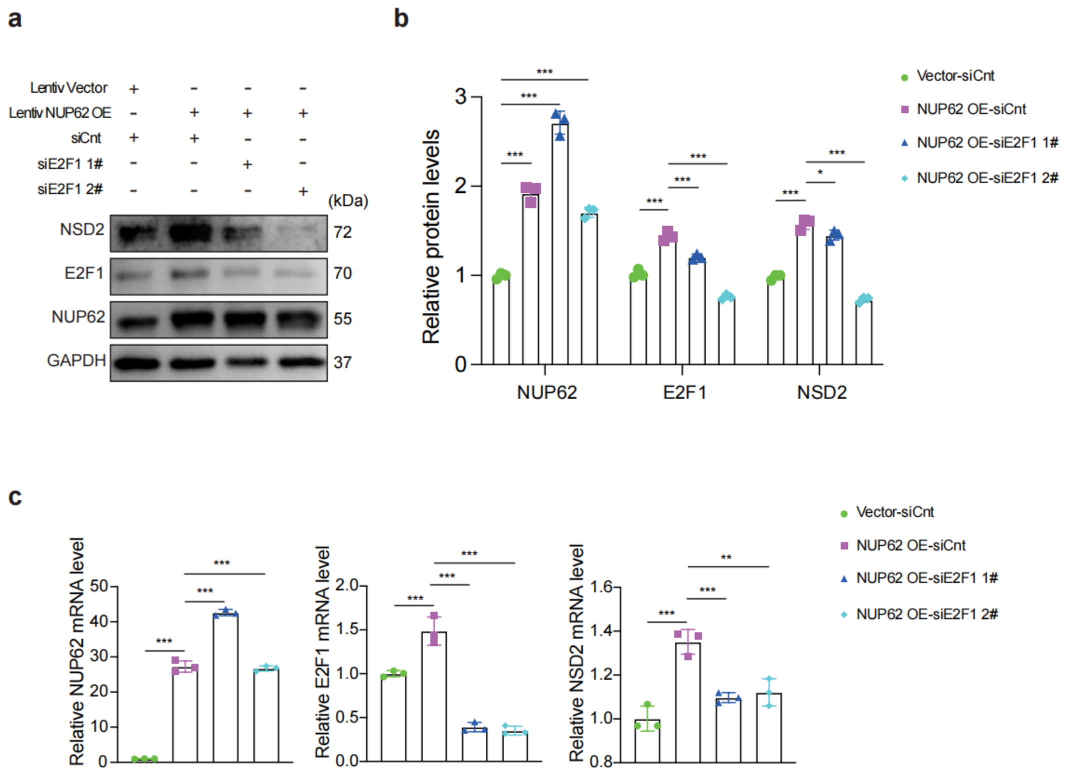

Fig. S10 NUP62 affects NSD2 transcription by regulating E2F1. a, b Western blotting for E2F1, NSD2, NUP62 and GAPDH was performed using lysates from HDPSCs transfected with lentiviral vector or overexpressing NUP62 and siRNA control or E2F1 siRNA. **c** Relative mRNA levels of NUP62, E2F1 and NSD2 in

HDPSCs transfected with the lentiviral vector or overexpressing NUP62 and siRNA control or E2F1 siRNA (n = 3). *$P<0.05$, **$P<0.01$ and ***$P<0.001$.

Supplemental Table S1. Antibodies for flow cytometry

Reagent or Resource	Source	Identifier
Anti-Isotype control antibody	Abcam	ab91357
Anti-CD73 antibody	Abcam	ab133582
Anti-CD146 antibody	Abcam	ab75769
Anti-CD105 antibody	Abcam	ab231774
Anti-human CD90 antibody	Biolegend	328109
Anti-human CD24 antibody	Biolegend	311103
Anti-human CD34 antibody	Life	12-0349-42
Anti-CD45 antibody	Biolegend	103112
Anti-CD29 antibody	Abcam	ab179471
Anti-CD44 antibody	Abcam	ab23396

Supplemental Table S2. The information of sh/siRNA sequences

Name	Source	The target sequence of shRNA
sh-scramble-NUP62	OBiO	CCTAAGGTTAAGTCGCCCTCG
shNUP62-1	OBiO	GTTCACGTTTGGCACTGCAAA
shNUP62-2	OBiO	ACCGGCTTTGCCTTGAATTTA
**Name**	**Source**	**The target sequence of siRNA**
siRNA Control	RIBOBIO	TTCTCCGAACGTGTCACGT
siRNA NSD2 1#	RIBOBIO	GGTCCAAAGTGTCGGGTTA
siRNA NSD2 2#	RIBOBIO	GGGCATTGTTCAAGCAGAA
siRNA Control	RIBOBIO	TTCTCCGAACGTGTCACGT
siRNA E2F1 1#	RIBOBIO	GAGACCTCTTCGACTGTGA
siRNA E2F1 2#	RIBOBIO	CTATGAGACCTCACTGAAT

Supplemental Table S3. RT–qPCR primers

Gene	Sequence 5’ to 3’
GAPDH-F1	GGAGCGAGATCCCTCCAAAAT
GAPDH-R1	GGCTGTTGTCATACTTCTCATGG
P16-F1	TGCCCAACGCACCGAATAGT
P16-R1	CAGCAGCTCCGCCACTCG
P21-F1	TGTCCGTCAGAACCCATGC
P21-R1	AAAGTCGAAGTTCCATCGCTC

P53-F1	AATCTACTGGGACGGAACAGCTTTGAGG
P53-R1	GGAGAGGAGCTGGTGTTGTTGGG
IL6-F1	GCCCAGCTATGAACTCCTTCT
IL6-R1	GAAGGCAGCAGGCAACAC
IL8-F1	ACTGAGAGTGATTGAGAGTGGAC
IL8-R1	AACCCTCTGCACCCAGTTTTC
MMP13-F1	ACTGAGAGGCTCCGAGAAATG
MMP13-R1	GAACCCCGCATCTTGGCTT
ALP-F1	GTGAACCGCAACTGGTACTC
ALP-R1	GAGCTGCGTAGCGATGTCC
RUNX2-F1	CAGACCAGCAGCACTCCATAT
RUNX2-R1	CAGCGTCAACACCATCATTC
BMP2-F1	ACTACCAGAAACGAGTGGGAA
BMP2-R1	GCATCTGTTCTCGGAAAACCT
DSPP-F1	GCATTCAGGGACAAGTAAGCA
DSPP-R1	CTTGGACAACAGCGACATCCT
DMP1-F1	AGCCATTCTGAGGAAGACGA
DMP1-R1	TGTTGTGATAGGCATCAACTGTTA
SOX2-F1	GCCGAGTGGAAACTTTTGTCG
SOX2-R1	GGCAGCGTGTACTTATCCTTCT
S100B-F1	TGGCCCTCATCGACGTTTTC
S100B-R1	ATGTTCAAAGAACTCGTGGCA
MAP2-F1	TCGTAGAGGGACGGAAAATTGA
MAP2-R1	GCCGTTGGTGTAGGGGTTC
TUBB3-F1	GGCCAAGGGTCACTACACG
TUBB3-R1	GCAGTCGCAGTTTTCACACTC
CDK1-F1	AAACTACAGGTCAAAGTGGTAGCC
CDK1-R1	TCCTGCATAAGCACATCCTGA
PCNA-F1	CCTGCTGGGATATTAGCTCCA
PCNA-R1	CAGCGGTAGGTCGAAGC
FOXM1-F1	GGAGCAGCGACAGGTTAAGG
FOXM1-R1	GTTGATGGCGAATTGTATCATGG
HMGA1-F1	GCTGGTAGGGAGTCAGAAGGA
HMGA1-R1	TGGTGGTTTTCCGGGTCTTG
HMGA2-F1	ACCCAGGGGAAGACCCAAA
HMGA2-R1	CCTCTTGGCCGTTTTTCTCCA
NSD2-F1	ACCGCGAGTGTTCTGTGTTC
NSD2-R1	GTCGTGGCCGTTAAACTTCTG
E2F1-F1	ACGCTATGAGACCTCACTGAA
E2F1-R1	TCCTGGGTCAACCCCTCAAG

Supplemental Table S4. The promoter region sequences of NSD2

GCTAGAGTGCAGTGGCATGATCTCGGCTCACTGCAACCTCCACTTCCCGGGTTCAAGTGATTCTCC
TGCCTCAGCCTCCTGAATAGTTGCGATTACAGATGCCCGTCACTATGCCTGGCTAATTTTTGTATTT
TTTTTTAGTAGAGACGGGGTTTCACCATGTTAGCCAGGCTGGTCTCGAACTCCTGACCTCAGGCGA
TCCACCTGTCTCGGCCTTCCAAAGTGCTTGTATTAAAGGTGTGAGCCACCATGCCTGGCACTGGCC
GTAATTTTAAAAATTCATTTATTTGTTTGGTGATAGAGACGAGGTCTTGCTATGTTGCCCAGTCTG
GTCTCCAACTCCTGGGCTCAAGCAATCCTTTCACTTTGGCCTCCCACAGTGCTGGCTCACACTTCTC
ACAGGTGTGAGCCACTGGCCTTGGCTCCATTTTGAATTAATTTTTGTTAAGGGTGTAAAGTCTGTG
TCTAGCCTGATTTTTTTTTGCATGTGGACATCCAGTTGTTCCAGCACCATTTGTCGAAAAGACTGTC
TTTTCTCCATTCCATTTCCTAGAGTCAAGCTCAGCTGACTCCATTATGTGGTCTATATCCGGGTTCT
CTATTCTGTTCATGGATCTATTTGTGTATTTTTCCACCAGTACCACAATGTCTCAATTACTGTAGCC
TTATAGTAAGTCTTGCAGCAACCCTTGTTTTTTTGGGGGGGCCACAACTCAACCCATACAGGGGCC
CAGCACCTGTGAAGGGACAGCAGCGGAGGCGGCGCGGGAGAGGAAGAGGAGGTGAACTGTGGC
CACTCCTTGGTGGCTGGAGGTGGGTCCGGGGAGGCAGGACCTGGGCTGAGGTGTCGTTCTATAGC
CCGGGTAGCGCACTTTCACAAAGCTCTCCTTGACCAAACTCTCGCCAGGCTCCCCTGGGCCCGCTT
CTCAGCGAGGGCTCAAGCTGGGCCTGTAAAGCTCGAGCAGACACTAAGGCTCAAGGCCGAAGGT
GTTCCCGGCTCCCTGAACCTGCGTGACAGAAAACCCAAGGCCGCCGGCTCCGGCAGCACCTGGAG
GCAGGAGCCGAACTTCCATGGGCGGCGGGCAGCAAATCACACCGGCTTCAACCGCCCGGCCCGCC
CACTTTTTGCAGCGTCTGACTTCCCCGCTCGCCCGCGCCGCTTCCTCCTCCCTGTGCCGCACGC
AGTCGCCCGCGCCACCCCCTGAAGCCCGGCTCTACCGCAGCGGCGCTGGGGTAGCGCCCCTGGAC
CAGGCGTCCTCCCCTCGCGAGCGGTCCTCCCGCAGCCGCGCCCAGCTCCGGGTCGGACCCTCGCC
CGGGGGACTCGTTCACGCCCTGCGGCCGTTAGGCGGGAAGAGCACGACGCCCTGCCCTGCCCCGT
CGTGGCGTCTGACCCTCTCCGCCACGTCTGTTCTGAGGCTCCGGGCCCGCCACGGCGGGGCCTCTG
CGGCGGTTTCCCCACCCGTCCCCGCACTGCCGCTCCGCGAGGCGGACGCCGCTAGCAGCCTCCCG
CGGGCCCCAGGCCCCGCGCCCCTCTCACGGGACAGGAGCTTGGGGACCCGCGCGGGCGGGCCGC
GCGCCCAACGCCAGCCTCCGCGGCCGCGCGCCCACGGCTCGGGGGCGCCGGCTCCGGACGGTGAT
TGGGCGCGGATCGTGACGTCACTGAGCGCGGCGCCCGCGGGCGCCGGGTCTGGGCCGGGTGCGC
GCCGCCGAGTAGCGGGAGCAGGGCGCGCGCCCGGCGCTACTCTGAGGCGGCTCGCGGGGCCCGC
GGCCCGGCCCTCCCGGCGCTGCCAGCCCCGCCCGCCCGCGCGGCCCCCACCCGCCGCGTGCCTGC
CCGCCCCGCCCGTGCGCGGCCCCGGCGCGGATTTGAAAAGCCCGGTCCGCGGGCCCCGCGAGCGC
GGCAGCCAATCAGCGGCGCGCACTTTTCCCGCGGCTTCTGCGAGGCGGCGGCGGCCCCGGCTGCG
GCGGCGGCGGCGGGCGGGAGGCGCGGGGGCGGGGTCGGCGCCGCGCGCGAGAGCCTCGGCCTGG
CCGCGCTGCGCCCGCCGCCGCCGCCGCCCCCTCCCCGCCTGGGCCCTACCGCCGCACGGCCCCGG
CCCCCTCCCAGCCTGCCGCTCCGGAGAGCCGCCCGCCGAGGATGCGACGCACCGCAGGTCACTGG
GGCGCCCGCCCAACAGTCGCGGGCCGCCAACCGCCGGGGCTG

Supplemental Table S5. Primers for Chip qPCR

Gene	Sequence 5’ to 3’
SIRT6-F1	CCGACAGGGTGACCACAGA
SIRT6-R1	GGCTAGACAATGGGAGGGAC

HMGA1-F1	GGCGGGTAGGCAAAGTGTCG
HMGA1-R1	CACAGCAAGTTGGCGAGCAG
HMGA2-F1	GCCCTCCCGACAAAGAACG
HMGA2-R1	CCCTGGCTACCCTGCACTCT
NSD2-F1	GGACAGGAGCTTGGGGACC
NSD2-R1	AGTGACCTGCGGTGCGTC
